# First Phylogeny of *Pseudolychnuris* Reveals Its Polyphyly and a Staggering Case of Convergence at the Andean Paramos (Lampyridae: Lampyrini)

**DOI:** 10.3390/insects13080697

**Published:** 2022-08-03

**Authors:** Angie Gisseth Ladino Peñuela, Juan Pablo Botero, Luiz Felipe Lima da Silveira

**Affiliations:** 1 Grupo de Investigación en Sistemática Molecular, Maestría en Ciencias-Entomología, Universidad Nacional de Colombia, Sede Medellín, Calle 59A No. 63-20, Medellín 050034, Colombia; aladino@unal.edu.co; 2 Grupo de Sistemática Molecular, Laboratorio de Entomología, Pontificia Universidad Javeriana, Carrera 7 40-62, Bogotá 11001000, Colombia; jp_bot@yahoo.com; 3 Biology Department, Western Carolina University, 1 University dr, Cullowhee, NC 28723, USA

**Keywords:** Andes, Andean region, Photinini, *Photinus*

## Abstract

**Simple Summary:**

Two species of Andean-endemic fireflies are herein revised, and their evolutionary relationships addressed for the first time. We show that despite their similarities, these two species are distantly related. We provide original reports of these species interacting with Andean-endemic flowers for the first time, and we propose that their similarities are due to participation in mimicry rings.

**Abstract:**

South America is likely the cradle of several New World firefly lineages but remains largely understudied. Despite several advances in firefly systematics in the Neotropical region, the Andean region has been largely unstudied for over a century. The Colombian Páramos are a critically threatened biodiversity hotspot that houses several endemic species, including the firefly genus *Pseudolychnuris*, with two species—*P. vittata* and *P. suturalis*. Here, by analyzing the phylogenetic relationships of *Pseudolychnuris*, we found that this genus is polyphyletic. *Pseudolychnuris vittata* and *P. suturalis* were found to be distantly related despite the striking similarity in outline and color pattern of males and females. We redescribe *Pseudolychnuris* and its type species *P. vittata*. Moreover, we revalidate *Alychnus* Kirsch, 1865 **stat. rev.** to accommodate *A. suturalis* **comb. nov.**, also redescribed here. We provide updated distribution maps and report field observations for both monotypic genera. Since adults visit flowers and interact with pollen and nectar, *Pseudolychnuris* and *Alychnus* may be occasional pollinators of Andean-endemic plants, a phenomenon previously neglected. Our findings reveal an interesting case of convergence between *Pseudolychnuris* and *Alychnus*—probably associated with life in the Páramos—and shed light on character evolution in the Photinini lineage of fireflies.

## 1. Introduction

Roughly a fourth of the 2200 firefly species are endemic to South America [1], the hypothesized cradle of several lampyrid lineages [2]. In fact, South America is home to several endemic genera of fireflies across its biomes. For example, recent systematic efforts shed light on several lineages of fireflies endemic to the Atlantic Rainforest [3,4,5,6,7,8], a rather common trend in soft-bodied beetle families [9,10,11,12]. Likewise, endemic genera were recently identified in the Amazon Rainforest [13] and in the Chilean *matorral* (*Cladodes* s.s. sensu [8]). Narrow endemicity is also widespread at the species level in South American fireflies [14,15]. Despite their singularity, a historical lack of targeted sampling, as well as taxonomic expertise and workforce, have hampered firefly studies in South America, compared to the current standing in other continents [16].

Targeted sampling recently conducted at the Serra dos Órgãos mountain range, in the Atlantic Rainforest, has identified this area as one of the “hottest spots” for firefly diversity worldwide [17]. The striking environmental heterogeneity found across its ~2200 m of elevation, and relatively mild seasonality, have been associated with high species turnover in the region, hence high species richness. Other Neotropical mountainous areas, with similar ecological properties, also yield a high richness of firefly species, particularly the Hispaniola [18,19,20] and the Los Tuxtlas biosphere reserve [21]. Therefore, it is reasonable to predict that the tropical Andes, the most critical biodiversity hotspot [22] and an important evolutionary species cradle [23,24], will also yield an outstanding diversity of firefly taxa. In fact, the environmental heterogeneity across the elevational and latitudinal range of the Andes are known drivers of its astonishing biodiversity [25,26,27,28]. Nevertheless, taxonomic studies on the Lampyridae across the Andes have been lacking for nearly one hundred years, making this region a frontier in the study of firefly diversity and evolution.

The Paramo is a grassland–shrubland Andean ecosystem found between approximately 3000 and 5000 m.a.s.l. from Venezuela to Northern Peru [29]. They are typical of the South American transition zone (sensu [30,31]) and are located in the upper part of the mountains between the Andean Forest strip and below the glaciers where these are present [32]. Colombia contains 50% of the páramos of the Earth and they occupy 2.5% of the Colombian territory [32,33,34]. Nearly 10% of Colombia’s biodiversity dwells in the Paramos, and a large number of endemic species (e.g., 3379 species of plants, 70 of mammals, 154 of birds, and 90 of amphibians) exist there [32,35].

Photinini LeConte, 1881, is the largest tribe of the Lampyridae: Lampyrinae, with nearly 750 species and over 30 genera [1,36]. Since the last comprehensive taxonomic catalog [1], several new genera and dozens of species were described [4,18,37,38,39], although few studies were based on phylogenetic analyses e.g., [7,13,40]. Recent phylogenetic studies underlined the value of phylogenetic analyses in understanding character evolution in Photinini, as well as in setting the framework for a revised classification of the tribe [7,13,40]. Despite recent advances, most Photinini taxa sorely need taxonomic reviews based on phylogenetic analyses. 

*Pseudolychnuris* Motschulsky, 1853, for example, has never been studied since its original description. The two species currently listed in *Pseudolychnuris*—namely *P. vittata* Motschulsky, 1853, and *P. suturalis* Motschulsky, 1853—have very similar overall morphology: outline parallel-sided, color pattern black with yellow stripes on elytra, as well as brachypterous and presumably flightless females [2]. Here, to test the monophyly of *Pseudolychnuris*, and explore its relationship among the Photinini, we ran phylogenetic analyses of morphological data contrasting the results of maximum parsimony analyses and Bayesian inference. 

## 2. Materials and Methods

### 2.1. Morphology and Terminology

Our study included materials from the following institutions: AGROSAVIA, Colección Taxonómica Nacional de Insectos “Luis Maria Murillo”, Bogotá, Colombia (CTNI; V. Vergara Navarro); Instituto Alexander von Humboldt, Villa de Leyva, Colombia (IAVH; J. Neita Moreno); Facultad de Agronomía de la Universidad Nacional, Bogotá, Colombia (UNAB; F. Serna Cardona); Instituto de Ciencias Naturales de la Universidad Nacional, Bogotá, Colombia (CNI; G. Amat); Museo de Historia Natural de la Pontificia Universidad Javeriana, Bogotá, Colombia (MPUJ; D. Forero); National Museum of Natural History, Washington, D.C., United States of America (USNM; M. Branham). 

For dissections, entire specimens or just their abdomen, depending on available material and permission from curators, and soaked in 10% KOH to digest muscles and clear the exoskeleton. Specimens were examined and imaged under a Leica M205 C stereomicroscope, using the Leica Application Suite X Automontage software. We follow the classification of Martin et al. [41] and the anatomical terminology of Silveira et al. [13].

We recorded label data for all type specimens using the following conventions: double quotes (“ ”) for label data quoted verbatim, double forward slashes (//) to separate labels; and brackets [ ] to enclose our comments or notes. All labels are typed unless otherwise noted.

Distribution maps of the species were made using R, with the packages “raster” [42], “sf” [43], “dplyr” [44], and “ggplot2” [45].

### 2.2. Phylogenetic Analyses

To test the monophyly of *Pseudolychnuris* in the context of its tribe, Photinini, we ran phylogenetic analyses comparing two approaches: maximum parsimony analysis and Bayesian inference. Our taxon sampling included 20 Lampyrinae taxa, in addition to one incertae sedis taxon (*Vesta thoracica* Olivier, 1790) morphologically close to one of our ingroup taxa, *Dilychnia guttula* (Fabricius, 1801) (see [40]). The tree was rooted in the lampyrini *Lampyris noctiluca* Linnaeus, 1758. The ingroup included the remaining 20 taxa were all Photinini, including three incertae sedis taxa (*Ethra marginata*, *Haplocauda albertinoi* Silveira, Lima, and McHugh, 2022, *Scissicauda disjuncta* (Olivier, 1896)), and 17 representing all four subtribes: Phosphaenina (*Phosphaenus hemipterus* (Geoffroy, 1785), *Phosphaenopterus metzneri* Schaufuss, 1870), Lucidotina (*Costalampys delicata* Silveira, Roza, Vaz and Mermudes, 2021; *Lucidota atra* (G. Olivier, 1790) and *L. banoni* Laporte, 1833; *Luciuranus josephi* Silveira, Khattar and Mermudes, 2016, and *L. sinistrus* Silveira, Khattar and Mermudes, 2016; *Pseudolychnuris vittata* Motschulsky, 1854, and *P. suturalis* Motschulsky, 1854; *Uanauna angaporan* Campello-Gonçalves, Souto, Mermudes and Silveira, 2019), Dadophorina (*Dadophora hyalina* (E. Olivier, 1907)), Photinina (*Photinus pyralis* (Linnaeus, 1767)), *P. corruscus* (Linnaeus, 1767); *Ybytyramoan praeclarum* Silveira and Mermudes, 2014). The material examined for this study is given below (see Taxonomy, below), and additional material used in the phylogenetic analyses is provided in Supplemental Material File S1. 

We coded 93 morphological characters for 21 taxa, using MESQUITE 3.61 [46]. We drew or modified 73 characters from Silveira et al. [13], following the guidelines of Sereno [47]. Measurement-based characters were taken at the longest or the widest point of the respective structure. Key character states are labeled in figures, abbreviated as C:S, where C and S indicate character and state number, respectively.

We implemented a maximum parsimony analysis using implied weights, a method where characters are weighted according to their homoplasy and, thus, characters with less homoplasy have more weight [48,49]. The K-value determines how strongly the analyses will weigh against homoplasy; however, there are no well-justified criteria to choose some particular value of K, and this decision is probably matrix-dependent [48]. To choose the value of K used in our analysis, we used the methodology proposed by Mirande [50], which divides at regular intervals the mean fit values of each character in the most parsimonious trees obtained under different values of K and for which the main criterion for choosing among these trees is their stability. This method was applied using the script for TNT developed by Mirande [50] (available at: http://phylo.wikidot.com/tntwiki, accessed on 1 January 2022). The most stable trees are those that share the largest number of nodes with the remaining trees, as measured by the SPR distance [49]. The best K range for the data matrix presented was in the intervals five to nine (Supplementary Materials Table S1); this range was chosen based on comparison and selection of the highest similarity coefficient (SPR). In each of these intervals, a single tree was obtained, and in all the intervals the tree obtained was the same, (see Supplementary Materials Table S1). We implemented symmetric resampling with default settings and 1000 replicates to estimate node support [51]. Consistency [52] and retention [53] indices are given for each character.

For the Bayesian inference, a model selection ran by ModelFinder [54] in IQTREEE2 [55] selected the MKV model [56] with equal state frequencies, 4 gamma categories, and correction for ascertainment bias (i.e., MK + FQ + ASC + G4) (Supplementary Materials File S2). The Bayesian inference was performed in MrBayes 3.2.7a [57,58] on XSEDE via the CIPRES Science Gateway V. 3.3 (phylo.org). The analysis ran 4 runs of 4 chains and 10^7^ generations, saving trees every 2000 generations, and discarded the first 25% as burn-in. The resulting trees were checked for convergence in Tracer v1.6 [59].

## 3. Results

### 3.1. Character List

Our matrix included characters from male specimens, spanning the three tagmata: head (9), thorax (29), and abdomen (66, 39 of which from the aedeagus) (Supplementary Materials File S3). For each character, the following is indicated: the number of steps (L); the consistency index (CI); and the retention index (RI).

1 Antenna, antennomeres III–IX, core, shape: (0) serrate, (1) cylindrical. L = 6; CI = 16; RI = 16.
2 Antenna, antennomeres III–IX, single lamellae: (0) absent, (1) present (branch longer than core antennomere). L = 3; CI = 33; RI = 0.
3 Clypeus, connection to frons: (0) connected by membrane throughout, (1) completely obliterate, (2) connate by median 1/3. L = 4; CI = 50; RI = 66.
4 Mandibles, orientation in frontal view: (0) overlapping, (1) crossed, (2) convergent. L = 2; CI = 100; RI = 100.
5 Mandible, apex, shape: (0) sharp, (1) blunt, (2) rounded. L = 3; CI = 66; RI = 50.
6 Labrum, sclerite, anterior margin, shape: (0) straight, (1) emarginate. L = 5; CI = 20; RI = 50.
7 Labium, submentum, anterior margin, shape: (0) straight, (1) notched. L = 1; CI = 100; RI = 100.
8 Labium, submentum, lateral margins, shape: (0) subparallel to slightly convergent posteriorly, (1) abruptly constrained posteriorly, (2) strongly convergent posteriorly. L = 11; CI = 18; RI = 25.
9 Labium, palp, palpomere III, lateral margins, shape: (0) obconical, (1) subparallel, (2) divergent apically. L = 5; CI = 40; RI = 40.
10 Pronotum, anterior margin, shape: (0) acuminate anteriorly, (1) evenly rounded. L = 3; CI = 33; RI = 33.
11 Pronotum (lateral view), anterior expansion, curvature: (0) curved upwards, (1) straight. L = 1; CI = 100; RI = 100.
12 Pronotum, lateral margin, length relative to disc: (0) less than 1/3, (1) nearly half, (2) at least 1. L = 4; CI = 25; RI = 50.
13 Pronotum, disc, sagittal depression: (0) absent, (1) present. L = 1; CI = 100; RI = 100.
14 Pronotum, by the disc, posterior margin, shape: (0) strongly sinuose, (1) almost straight. L = 1; CI = 100; RI = 100.
15 Hypomeron (lateral view), ratio between hypomeron depth and pronotal lateral expansion width: (0) approximately as long, (1) at least a 1/5 shorter, (2) at least a 1/5 longer. L = 4; CI = 25; RI = 62.
16 Pronotum, posterior corner, notch, presence: (0) absent, (1) present. L = 7; CI = 28; RI = 44.
17 Hypomeron (ventral view), area anterior to prosternal insertion, shape: (0) projecting outwards, (1) straight. L = 1; CI = 100; RI = 100.
18 Prosternum, anterior margin, shape: (0) medially sinuose, (1) straight. L = 5; CI = 20; RI = 50.
19 Mesoscutellum, posterior margin, shape: (0) rounded, (1) truncate. L = 4; CI = 25; RI = 40.
20 Elytron, outer margin, shape: (0) straight, (1) rounded, (2) convergent posteriorly. L = 7; CI = 42; RI = 33.
21 Wing, position of MP_3+4_ split, relative to CuA_1_: (0) more basal, (1) more apical. L = 4; CI = 25; RI = 57.
22 Wing, AA3, shape: (0) short (almost as long as wide) and almost perpendicular to AA4, (1) elongate and with an acute angle to AA4. L = 3; CI = 33; RI = 60.
23 Wing, r3: (0) absent, (1) present. L = 2; CI = 50; RI = 50.
24 Proleg, anterior claw, tooth: (0) absent, (1) present. L = 3; CI = 33; RI = 33.
25 Proleg, tibial spurs, count: (0) zero, (1) one, (2) two. L = 9; CI = 22; RI = 36.
26 Mesoleg, anterior claw, tooth: (0) absent, (1) present. L = 3; CI = 33; RI = 33.
27 Mesoleg, tibial spurs, count: (0) zero, (1) one, (2) two. L = 7; CI = 28; RI = 50.
28 Metaleg, tibial spurs, count: (0) zero, (1) one, (2) two. L = 7; CI = 28; RI = 50.
29 Tergum I, laterotergite, shape: (0) indistinct, (1) triangular, (1) trapezoidal, (1) quadrangular. L = 5; CI = 60; RI = 60.
30 Tergum I, spiracle, shape: (0) reniform, (1) subcircular. L = 1; CI = 100; RI = 100.
31 Tergum VII, posterior angles, shape: (0) projected, embracing anterior angles of pygidium, (1) rudimentary, slightly projected backwards. L = 1; CI = 100; RI = 100.
32 Sterna II–VIII, width variation: (0) progressively narrow, widest by sterna III–IV, (1) widest by sternum V. L = 4; CI = 50; RI = 60.
33 Sternum VI, lantern: (0) absent, (1) present. L = 4; CI = 25; RI = 40.
34 Sternum VII, lantern: (0) absent, (1) present. L = 3; CI = 33; RI = 33.
35 Sternum VIII, length relative to VII: (0) as long as, (1) slightly longer, (2) at least a fifth shorter, (3) 2× as long, (4) at least 3× longer. L = 4; CI = 50; RI = 83.
36 Sternum VIII, lateral margins, shape: (0) rounded, (1) divergent up to basal 1/4, then convergent posteriorly. L = 2; CI = 50; RI = 0.
37 Sternum VIII, posterior margin, shape: (0) almost straight, (1) sinuose. L = 9; CI = 22; RI = 36.
38 Sternum VIII, posterior margin, median projection: (0) absent, (1) present. L = 6; CI = 16; RI = 37.
39 Sternum VIII, posterior margin, median projection, shape: (0) tiny, (1) elongate, (2) wide, triangular. L = 2; CI = 100; RI = 100.
40 Pygidium, shape: (0) at least a fifth wider than long, (1) as long as wide, (2) at least a fifth longer than wide. L = 6; CI = 33; RI = 66.
41 Pygidium, lateral margins, shape: (0) subparallel, (1) rounded, (2) divergent posteriorly convergent posteriorly. L = 4; CI = 75; RI = 66.
42 Pygidium, posterior margin, central 1/3, shape: (0) almost straight, (1) rounded, (2) emarginate, (3) medially notched. L = 7; CI = 42; RI = 42.
43 Pygidium, posterolateral corners, degree of development: (0) well-developed, (1) barely conspicuous. L = 6; CI = 16; RI = 44.
44 Pygidium, posterolateral corners, length relative to central 1/3: (0) shorter, (1) as long as, (2) longer. L = 11; CI = 18; RI = 30.
45 Syntergite, shape (proportion): (0) longer than wide, (1) wider than long. L = 4; CI = 25; RI = 0.
46 Syntergite, lateral margin, shape: (0) convergent posteriorly, (1) subparallel. L = 2; CI = 50; RI = 66.
47 Syntergite, anterior margin, shape: (0) mildly emarginated, (1) strongly indented, (2) almost straight. L = 4; CI = 50; RI = 71.
48 Syntergite, pattern of sclerotization: (0) evenly sclerotized, (1) completely split in the middle, forming two plates. L = 1; CI = 100; RI = 100.
49 Syntergite, length relative to sternum IX: (0) 1/3, (1) 1/2, (2) 2/3, (3) 1/5. L = 11; CI = 27; RI = 11.
50 Syntergite, posterolateral corners, chaetotaxy: (0) glabrous, (1) covered in setae, (2) with dome-shaped sensillae. L = 8; CI = 25; RI = 60.
51 Sternum IX, lateral rods, shape: (0) subparallel, (1) evenly convergent, (2) abruptly convergent, (3) biconcave (apically divergent). L = 3; CI = 66; RI = 0.
52 Sternum IX, lateral rods, tips, connection: (0) separated, (1) fused. L = 5; CI = 20; RI = 42.
53 Sternum IX, length relative to aedeagus (including phallobase): (0) slightly shorter, (1) slightly longer, (2) a 1/3 longer. L = 8; CI = 37; RI = 58.
54 Sternum IX, position relative to VIII: (0) completely covered, (1) partially exposed. L = 1; CI = 100; RI = 100.
55 Sternum IX, posterior half, degree of excavation: (0) evenly sclerotized, (1) medially membranous, (2) emarginated, (3) deeply clefted (to at least a fifth sternum length). L = 6; CI = 50; RI = 50.
56 Phallobase, bilateral symmetry: (0) symmetrical, (1) asymmetrical. L = 3; CI = 33; RI = 33.
57 Phallobase, length relative to phallus: (0) at least a fourth shorter, (1) as long as, (2) at least a fourth longer. L = 8; CI = 25; RI = 50.
58 Phallobase, sagittal line: (0) absent, (1) present. L = 4; CI = 25; RI = 66.
59 Phallobase, sagittal line, extension: (0) throughout phallobase, (1) not reaching apical margin. L = 3; CI = 33; RI = 50.
60 Phallobase, apical margin, shape: (0) slightly emarginate, (1) deeply emarginate (C-shaped), (2) medially clefted. L = 8; CI = 25; RI = 33.
61 Phallus, dorsal plate, median connection to parameres: (0) connected by membrane, (1) connate fused. L = 3; CI = 66; RI = 66.
62 Phallus, dorsal plate, base, pattern of sclerotization: (0) evenly sclerotized, (1) widely membranous. L = 1; CI = 100; RI = 100.
63 Phallus, struts, condition: (0) absent, (1) present (visible through the phallobase). L = 2; CI = 50; RI = 88.
64 Phallus, dorsal plate, ventrobasal processes, presence: (0) absent, (1) present. L = 1; CI = 100; RI = 100.

Remark: These have been called “ventrobasal process” (after Green [60]) or “dorsal excrescences” [61]. Since these structures are clearly projected towards the ventral side, we follow Green [60].

65 Phallus, dorsal plate, ventrobasal processes, shape (in ventral view): (0) thin, wider than long, (1) globose. L = 1; CI = 100; RI = 100.
66 Phallus, dorsal plate, ventrobasal processes, shape (in apical view): (0) divergent, (1) convergent. L = 1; CI = 100; RI = 100.
67 Phallus, dorsal plate, subcleft transverse groove: (0) absent, (1) present. L = 1; CI = 100; RI = 100.
68 Phallus, dorsal plate, length relative to parameres: (0) nearly a fifth longer, (1) at least a fifth shorter, (2) as long as, (3) twice as long. L = 7; CI = 42; RI = 20.
69 Phallus, dorsal plate, condition: (0) entire, (1) medially split. L = 1; CI = 100; RI = 100.
70 Phallus (dorsal view), dorsal plate, anterior margin, shape: (0) rounded, (1) pointed, (1) truncate, (1) clefted. L = 7; CI = 42; RI = 69.
71 Phallus, dorsal plate, degree of median indentation: (0) nearly a 1/3 plate length, (1) nearly 1/2 plate length, (2) nearly 2/3 plate length, (3) completely divided, (1) slightly emarginate. L = 2; CI = 100; RI = 100.
72 Phallus, dorsal plate, apical arms (of indented Phallus), shape: (0) widely separated and slightly convergent, (1) contiguous, (2) fused, (3) apically divergent. L = 12; CI = 25; RI = 18.
73 Phallus (lateral view), dorsal plate, overall shape: (0) straight, (1) bent dorsally, (2) slightly bent ventrally, (3) sinuose. L = 7; CI = 42; RI = 71.
74 Phallus, dorsal plate, basal abrupt constriction: (0) absent, (1) present. L = 1; CI = 100; RI = 100.
75 Phallus, dorsal plate, basal joint: (0) absent, (1) present. L = 3; CI = 33; RI = 0.
76 Phallus, dorsal plate, subapical outer teeth: (0) absent, (1) present. L = 2; CI = 50; RI = 0.
77 Phallus, dorsal plate, sides, texture: (0) smooth, (1) spiked. L = 1; CI = 100; RI = 100.
78 Phallus, dorsal plate, longitudinal window: (0) absent, (1) present. L = 1; CI = 100; RI = 100.
79 Phallus, dorsal plate, subapical keel: (0) absent, (1) present. L = 1; CI = 100; RI = 100.
80 Phallus, ventral plate: (0) absent, (1) present. L = 2; CI = 50; RI = 87.
81 Phallus, ventral plate, length relative to dorsal plate: (0) half as long, (1) as long as or slightly longer, (2) a 1/3 shorter. L = 5; CI = 60; RI = 50.
82 Phallus, endosac, opening, shape: (0) cylindrical, (1) cul-de-sac. L = 1; CI = 100; RI = 100.
83 Base of paramere, basal projection: (0) absent, (1) present. L = 1; CI = 100; RI = 100.
84 Paramere, subapical ventral spike, presence: (0) absent, (1) present. L = 2; CI = 50; RI = 75
85 Paramere, subapical ventral spike, shape: (0) pointy, (1) elongate. L = 1; CI = 100; RI = 100.
86 Paramere, subapical membranous appendage: (0) absent, (1) present. L = 1; CI = 100; RI = 100.
87 Paramere, apex, curvature in lateral view: (0) straight, (1) slightly curved ventrally, (2) evenly curved inwards, (3) curved dorsally, (4) embracing phallus ventrally, (5) sinuose, (6) strongly curved dorsally. L = 13; CI = 46; RI = 53.
88 Paramere, apex, sclerotization relative to core paramere: (0) as sclerotized, (1) distinctly membranous, (2) more sclerotized, darker. L = 4; CI = 25; RI = 50.
89 Paramere, apex (tip), shape: (0) wide, rounded, (1) peg-like, blunt, (2) pointed, (3) truncated. L = 5; CI = 40; RI = 57.
90 Paramere (lateral view), basal lobe: (0) absent, (1) present. L = 1; CI = 100; RI = 100.
91 Paramere (lateral view), basal lobe, shape: (0) robust, rounded, (1) narrow and acute, (2) rudimentary. L = 2; CI = 100; RI = 100.
92 Paramere, inner face, shape: (0) smooth, (1) excavate. L = 3; CI = 33; RI = 33.
93 Paramere, base, orientation relative to phallus: (0) dorsal, (1) lateral (coplanar). L = 4; CI = 25; RI = 57.

### 3.2. Pseudolychnuris Is Polyphyletic

**Figure 1:**
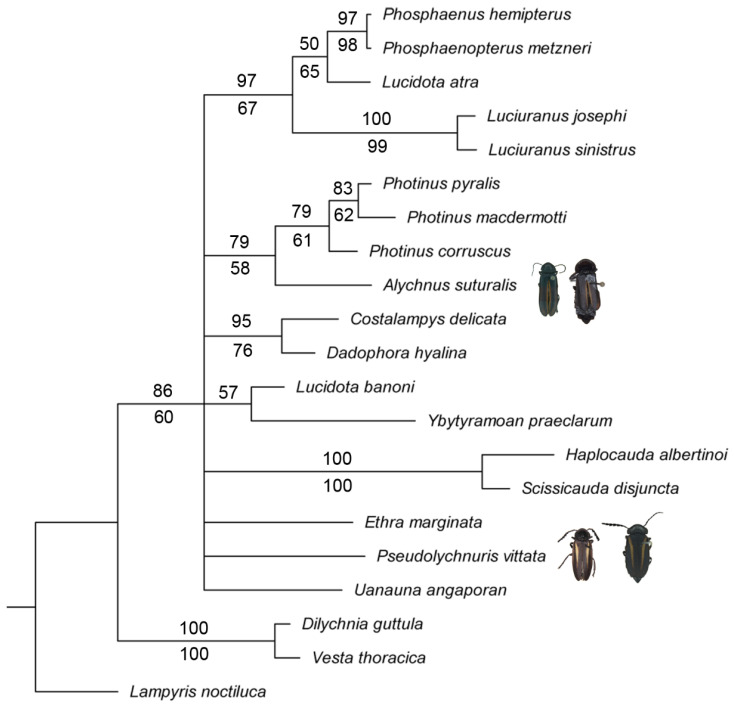
Bayesian phylogeny of *Pseudolychnuris*, based on 94 morphological characters of 22 taxa, showing that *P. suturalis* and *Alychnus suturalis* **comb. nov.** are not monophyletic. Values above branches: Bayesian posterior probabilities. Below branches: symmetrical resampling estimated from our preferred parsimony analysis with implied weights (K = 2.688). Values below 50 are omitted.

**Figure 2:**
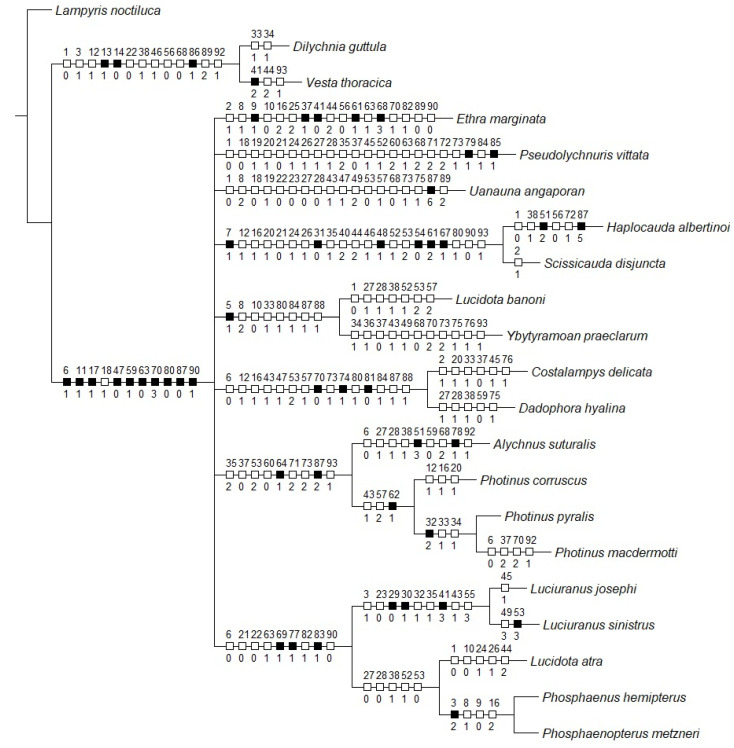
Uncontroverted apomorphies (black squares) and homoplasies (white squares) mapped on the Bayesian consensus tree. Values above branches: Character number. Below branches: character states.

The results of the Bayesian inference (BI) and the parsimony analysis (PA) were perfectly congruent (Figure 1). The only conflicting topologies observed in the better resolved PA had no support and varied across the range of *k* investigated. Both approaches recovered *Pseudolychnuris* as polyphyletic, since *P. vittata* and *P. suturalis* were never recovered as sister taxa (Figure 1). Instead, *P. suturalis* was consistently recovered as a sister to *Photinus*—represented by *P. corruscus* (*P. pyralis + P. macdemortti*)—with moderate support (Figure 1). As such, we herein revalidated *Alychnus* Kirsch, 1865 **stat. rev.**, previously in synonymy with *Pseudolychnuris* (see below), to include *A. suturalis* **stat. rev.** As recovered in the PA, apomorphies of *Alychnus* are the apically divergent lateral rods of the sternum IX: (51:3; Figure 15E,F), and the dorsal plate of the phallus with sides bearing teeth (78:1; Figure 15G–L) (Figure 2). Interestingly, two unambiguous synapomorphies support the sister–group relationship between *Alychnus* and *Photinus*, namely: the dorsal plate of the phallus bearing ventrobasal processes (65:1; Figures 7G and 15L), and the apex of the paramere evenly curved inwards (88:2; Figures 7G–K and 15G–L). Seven homoplasies also support this node (Figure 2).

*Pseudolychnuris* was recovered in a polytomy at a deeper split in both BI and the PA (Figure 1). The BA recovered, with moderate support, *P. vittata* in a polytomic node with *Uanauna*, *Ethra*, (*Ybytyramoan* + *Lucidota*), (*Dadophora* + *Costalampys*), (*Alychnus + Photinus*), and *Luciuranus* (*Lucidota atra* (*Phosphaenus + Phosphaenopterus*)). As per the PA, *Pseudolychnuris* is supported by one unambiguous apomorphy: the presence of a unique longitudinal window on the dorsal plate of the phallus (79:1; Figure 7G), in addition to 19 homoplasies (Figure 2).

**Figure 3:**
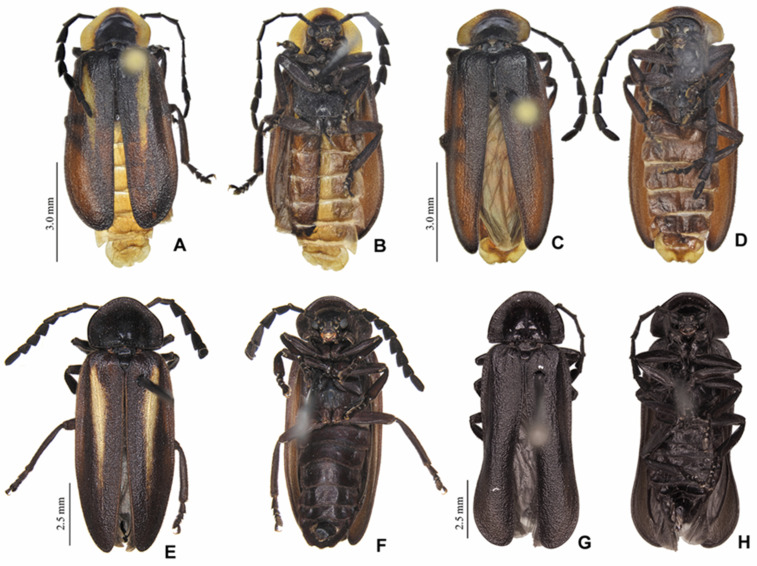
*Pseudolychnuris vittata* Motschulsky, 1854, males, chromatic variation. (**A**) dorsal, (**B**) ventral view, ♂-MPUJ. (**C**) dorsal, (**D**) ventral view, ♂-MPUJ. (**E**) dorsal, (**F**) ventral view ♂, IAVH-078002. (**G**) dorsal, (**H**) ventral view 1♂ IAvH-079001.

#### 3.2.1. Taxonomy

***Pseudolychnuris* Motschulsky, 1853**

**(** **Figure 3, Figure 4, Figure 5, Figure 6, Figure 7, Figure 8** **and Figure 9** **and Figure 11E–G)**

*Pseudolychnuris* Motschulsky, 1853: 32 [62]; Oliver, 1911: 70 [63]; Silveira et al., 2016: 376 [64]; Campello-Gonçalves et al., 2019: 66 [65]; Martin et al., 2019: 11 [41]; Ferreira et al., 2022: 236 [66].

**Type species:***Pseudolychnuris vittata* Motschulsky, 1854.

**Figure 4:**
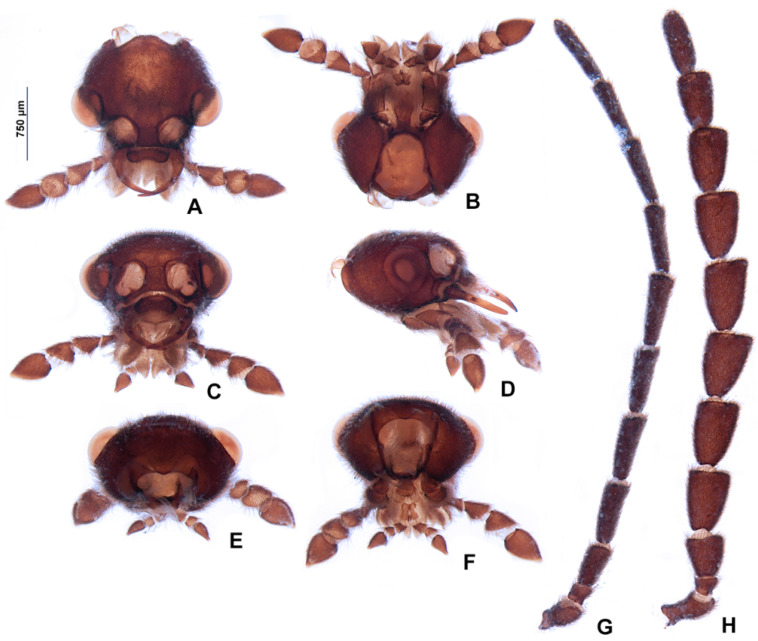
*Pseudolychnuris vittata* Motschulsky, 1854, male head, IAVH-078002. Head capsule (**A**–**F**): (**A**) dorsal, (**B**) ventral, (**C**) frontal, (**D**) lateral, (**E**) posterior, (**F**) occipital. Antenna (**G**,**H**): (**H**) lateral, (**G**) dorsal.

**Figure 5:**
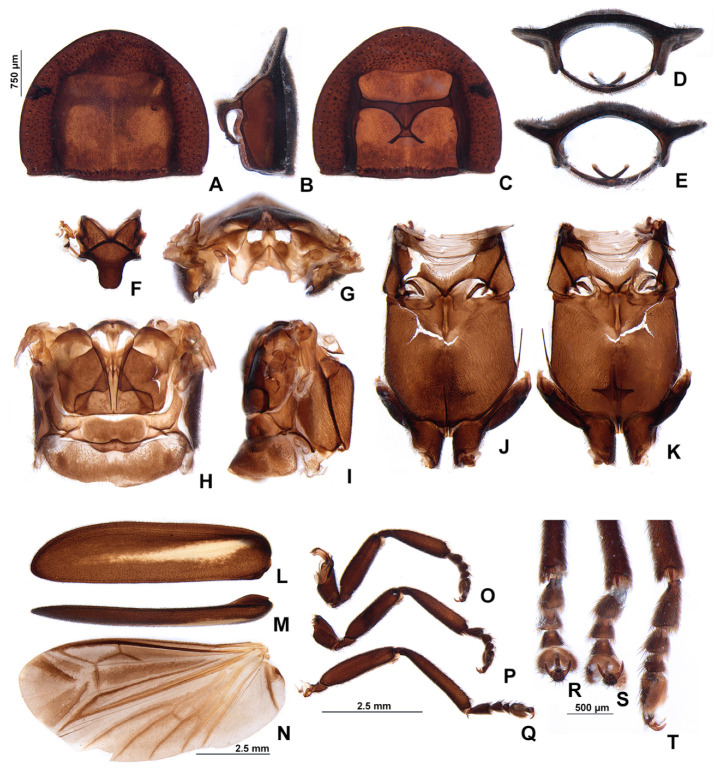
*Pseudolychnuris vittata* Motschulsky, 1854, male thorax, IAVH-078002. Prothorax (**A**–**E**): (**A**) dorsal, (**B**) lateral, (**C**) ventral, (**D**) anterior, (**E**) posterior. (**F**) Mesoscutellum, dorsal. Metanotum (**G**,**H**): (**G**) anterior, (**H**) dorsal. Pterothorax (**I**–**K**): (**I**) lateral, (**J**) dorsal, (**K**) ventral. Elytra (**L**,**M**): (**L**) ventral, (**M**) lateral. (**N**) Wing, dorsal. Outline of left legs (**O**–**Q**): (**O**) proleg, (**P**) mesoleg, (**Q**) metaleg. Detail of the inwards view of left legs (**R**–**T**)—note the tibial spurs: (**R**) proleg, (**S**) mesoleg, (**T**) metaleg.

**Figure 6:**
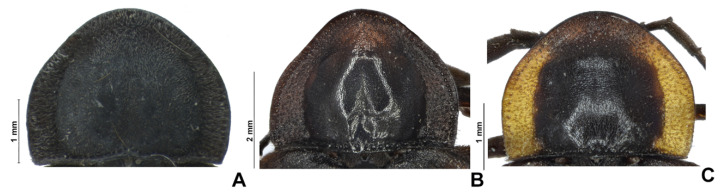
*Pseudolychnuris vittata* Motschulsky, 1854, male prothorax variation. (**A**) ♂, UNAB, (**B**) ♂, ICN-100862, (**C**) ♂, ICN-100863.

**Figure 7:**
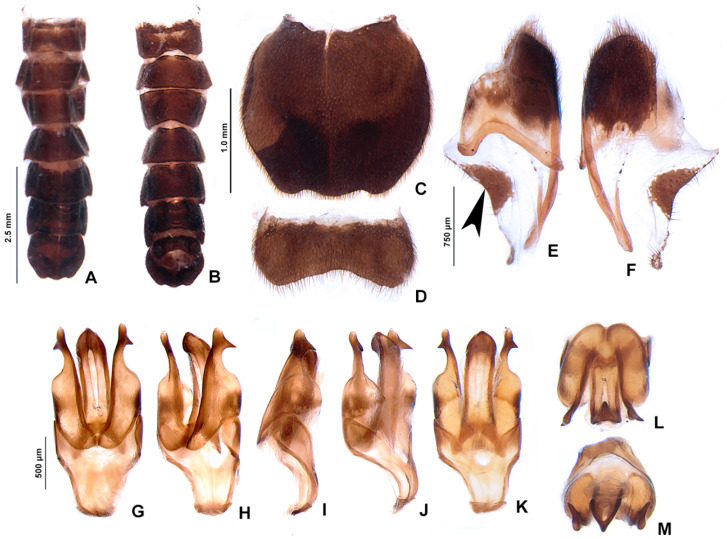
*Pseudolychnuris vittata* Motschulsky, 1854, male abdomen, IAVH-078002. Whole abdomen (**A**,**B**): (**A**) dorsal, (**B**) ventral. (**C**) Pygidium, dorsal. (**D**) Sternum VIII. Aedeagal sheath (**E**) Syntergite, dorsal**.** Black arrow shows triangular sclerite from beneath sternum VIII (**F**) Sternum IX, ventral. Aedeagus (**G**–**M**): (**G**) ventral, (**H**) ventro-lateral, (**I**) lateral, (**J**) dorso-lateral, (**K**) dorsal, (**L**) anterior, (**M**) posterior.

**Figure 8:**
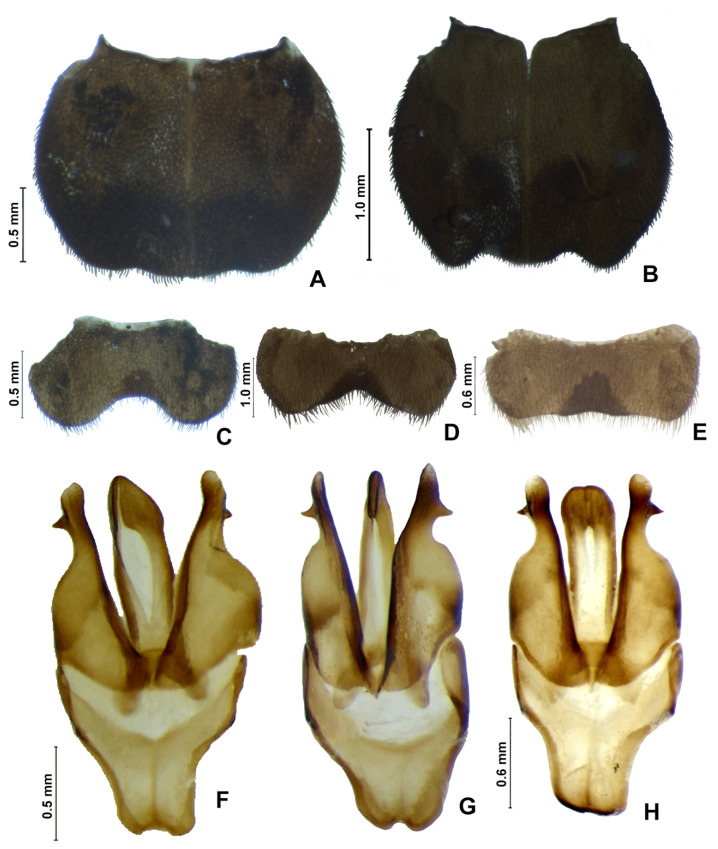
*Pseudolychnuris vittata* Motschulsky, 1854, male, abdomen variability. Pygidium, dorsal (**A**,**B**): (**A**) ♂, UNAB (**B**) ♂, ICN-100862. Sternum VIII (**C**–**E**): (**C**) ♂, UNAB, (**D**) ♂, ICN-100863, (**E**) ♂, ICN-100850. Aedeagus (**F**–**H**): (**F**) ♂, ICN-100862, (**G**) ♂, UNAB (**H**) ♂, ICN-100850.

**Figure 9:**
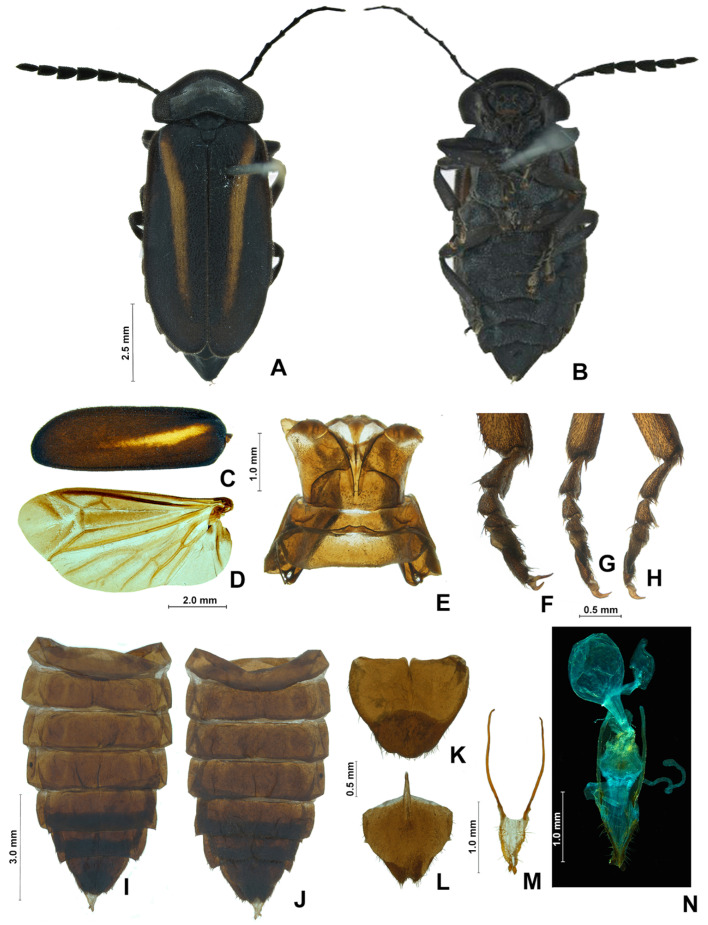
*Pseudolychnuris vittata* Motschulsky, 1854, female. (**A**) dorsal, (**B**) ventral, (**C**) elytra, ventral, (**D**) wing, dorsal, (**E**) pterothorax, dorsal. (**F**–**H**): (**F**) proleg, (**G**) mesoleg, (**H**) metaleg. Whole abdomen (**I**,**J**): (**I**) dorsal, (**J**) ventral. (**K**) Pygidium, dorsal. (**L**) Sternum VIII, (**M**) Ovipositor, (**N**) Internal anatomy of the reproductive tract.

**Diagnosis:** Antennae serrate (Figure 4G,H). Labrum with anterior margin slightly emarginate (Figure 4A), connected to frons by membrane throughout. Pronotum bearing a notch by the posterior angle (Figure 5A Tibial formula 1-1-1 (Figure 5R–T). Lanterns absent (Figure 7B). Sternum VIII with posterior margin slightly to moderately emarginate (Figure 7D and Figure 8C–E), lacking a median pointed projection. Sternum IX with lateral rods basally fused (Figure 7E,F). Phallobase with apical margin deeply clefted (Figure 7K). Phallus with dorsal plate bearing a lightly sclerotized longitudinal window (Figure 7G), ejaculatory duct running ventral to the dorsal plate (Figure 7M), basal struts well-developed (Figure 7G). Paramere with a basally elongate subapical spike, inner margin smooth (Figure 7G–K). Female brachypterous (Figure 9A,B), sternum VI with posterior margin straight (Figure 9J).

#### 3.2.2. Description

**Male. Head** capsule nearly a 1/3 wider than long (Figure 4A), vertex slightly convex (Figure 4C). Antennae serrate (Figure 4G,H), increasing in length up to antennomere VII, then decreasing toward apex. Mandibles overlapping (Figure 4C), apex acute. Labrum with anterior margin slightly emarginate (Figure 4A), connected to frons by membrane throughout. Gula 1/4 as long as submentum, 4× wider than long (Figure 4B). Submentum with anterior margin rounded, sides slightly convergent posteriorly. Occiput pyriform (Figure 4F). Labial palp with sides divergent, apex straight (Figure 4D,F).

**Thorax** with pronotum nearly 3× longer than elytron (Figure 3). Pronotum semilunar (Figure 5A and Figure 6), with a notch anterior to the posterior angle; disc convex (Figure 5D,E), lateral expansions as wide as 1/3-disc width and as wide as hypomeron depth (Figure 5B), anterior expansion 1/2 as long as disc (Figure 5A). Hypomeron nearly 2× longer than deep (Figure 5B). Prosternum with anterior margin almost straight (Figure 5C). Mesoscutellum with posterior margin subtruncate (Figure 5F). Elytron subparallel-sided, outer margin straight to slightly rounded (Figure 5L); marginal costa and epipleuron well developed (Figure 5M). Metanotum with anterior margin bisinuose (Figure 5G), scutum-prescutal ridge almost reaching posterior margin of alinotum (Figure 5H), metascutellum 1/4 as long as alinotum; metapostnotal plate with central 1/3 slightly emarginate (Figure 5H). Mesosternum–mesoespisternum suture obliterate (Figure 5J). Hind wing oblong, slightly beyond 2× wider than long (Figure 5N), R vein reaching anterior margin, AA3 elongate and at an acute angle with AA4, r3 present, MP_3+4_ split basal to CuA_1_. Pro and mesoleg with a rounded tooth by the anterior claw. Each leg with one tibial spur (Figure 5R–T). Proesdosternum well developed, apically rounded (Figure 5C–E), metaendosternum diamond-shaped (Figure 5k).

**Abdomen** with sternites II–IX visible (Figure 7A,B). Laterotergite subquadrangular (Figure 5I). Tergites II–VII with posterior angles poorly developed. Lanterns absent. Pygidium nearly as wide as long, posterior margin bisinuose, sides rounded, posterior angles rudimentary (Figure 7C). Sternum VIII 3× wider than long (Figure 7D and Figure 8C–E), nearly half as long as VII, with posterior margin slightly to moderately emarginate, lacking a median pointed projection, with a triangular sclerite fonded underneath. Syntergite wider than long and a 1/3 shorter than sternum IX, with anterior margin slightly emarginate, posterior corners bearing bristles (Figure 7E,F). Sternum IX slightly longer than aedeagus (Figure 7E,F), partially exposed under VIII posterior margin rounded, lateral rods basally fused. **Phallus** with phallobase slightly asymmetric (Figure 7G–L and Figure 8F–H), nearly 1/4 as long as phallus, sides emarginate, apical margin medially clefted; with a complete longitudinal keel. Phallus approximately as long as parameres (Figure 7G–K and Figure 8F–H); dorsal plate with well developed basal struts, with a longitudinal lightly sclerotized window, apex rounded to acute; ventral plate absent; ejaculatory duct running ventrally to dorsal plate (Figure 7M). Parameres connected to phallus by membrane (Figure 7G–K and Figure 8F–H), apically membranous, placed dorsal to phallus, ventral lobe present, acuminate; subapical spike present and longitudinally elongate.

**Female.** Antennomeres III-IX as in males, but slightly shorter (Figure 9A,B). Elytron brachypterous (Figure 9A), slightly shorter than that of male, leaving exposed at least the pygidium, sometimes tergites VII. Hind wing 2× wider than long. Metanotum with anterior margin bisinuose (Figure 5G). Leg claws without teeth (Figure 9F–H). Pygidium as long as wide, sides convergent posteriorly, posterior margin barely bisinuose (Figure 9K). Sternum VII slightly emarginate, VIII medially indented, sides convergent posteriorly, spiculum ventrale almost 1/3 as long as sternum, with posterior margin emarginate medially, deeper in sternite VIII. One spermatheca and one accessory gland present. Ovipositor with coxital rods 2× longer than core coxite.

**Figure 10:**
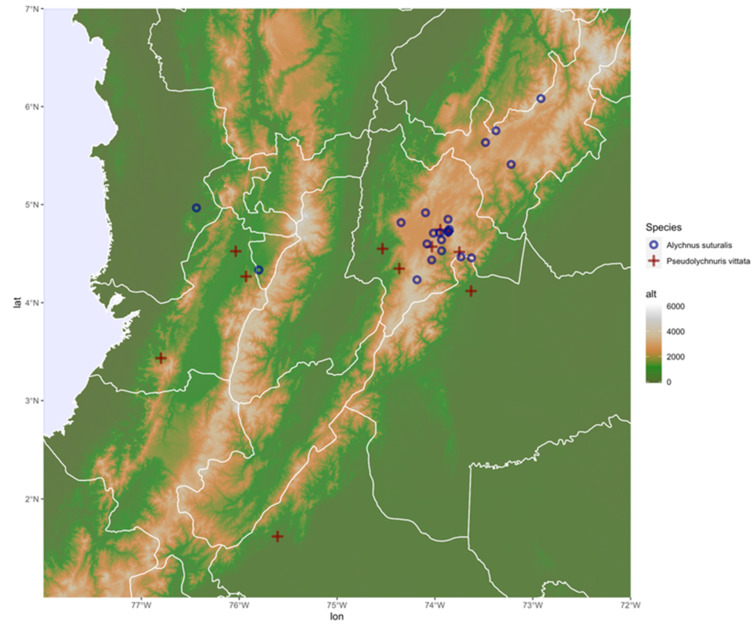
Distribution of *Alychnus suturalis* (Motschulsky, 1854) and *Pseudolychnuris vittata* Motschulsky, 1854.

**Distribution (based on *P. vittata*):***Pseudolychnuris* is endemic to the Colombian Andes, present in municipalities of the departments of Cundinamarca, Valle del Cauca, Meta, and Caquetá. Its altitude range goes from 550 to 3400 m.a.s.l. (Figure 10). Its location contrasted with data from Colombian ecosystems [67] suggests that *Pseudolychnuris* is found in humid mountainous and sub-humid high Andean landscapes, as well as in landscapes of erosional alluvial plains of Andean rivers in the department of Caquetá. Considering the material examined, adults of *Pseudolychnuris* are more commonly found between November and December and are mostly absent from March through July.

**Figure 11:**
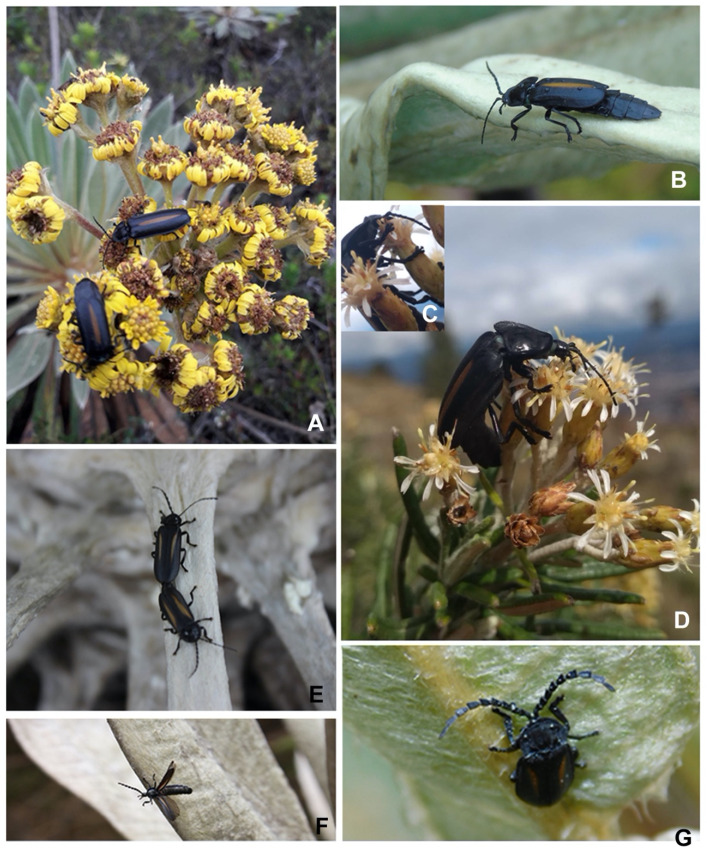
*Alychnus suturalis* and *Pseudolychnuris vittata* in situ. (**A**) 1♂ and 1♀ *A. suturalis*. Picture taken by L. Pirateque on 15 October 2018 at Parque Ecológico Matarredonda, Cundinamarca, Colombia. (**B**) ♀ *A. suturalis*. Picture taken by N. Silva on 18 August 2019 in Choachí, Cundinamarca, Colombia. (**C**,**D**) ♀ *A. suturalis*. Picture taken by A. Ladino on 15 November 2020 in La Calera, Cundinamarca, Colombia. (**E**) 1♂, 1♀ *P. vittata*. Picture taken by Diego Amaya on 9 December 2019 in Ubaté, Cundinamarca, Colombia. (**F**) 1♂ *P. vittata*. Picture taken by M. Gómez on 30 November 2013 at PNN Chingaza. (**G**) *P. vittata*. Picture taken by D. Amaya on 19 August 2019 in Choachí, Cundinamarca, Colombia.

**Remarks:***Pseudolychnuris* originally comprised two species: *P. vittata* and *P. suturalis*. Here, we showed that *P. suturalis* is more distantly related, and revalidated *Alychnus* Kirsch, 1865, to accommodate *A. suturalis* (Motschulsky, 1854) **comb. nov.** (see above). The phylogenetic affinities of *Pseudolychnuris* are still poorly known, since in our analyses (Figure 1), this genus appears in a polytomy. 

*Pseudolychnuris* is unique among Photinini by the presence of a lightly sclerotized longitudinal window on the dorsal plate of the phallus (79:1). Even though this window resembles the cleft on the dorsal plate of other Photinini, including *Alychnus* and *Photinus* species (e.g., Figure 15G), in *Pseudolychnuris,* the ejaculatory duct runs *ventral* to the dorsal plate. In contrast, the ejaculatory duct runs *through* the dorsal plate in *Alychnus* and *Photinus* species. Among the Photinini, *Pseudolychnuris* is most similar to *Alychnus* and “dark” (i.e., lantern-less) species of *Photinus*. However, *Pseudolychnuris* has a lack of ventrobasal processes on the dorsal plate of the phallus (65:0), typical of *Photinus* and *Alychnus* (65:1), and has spikes on the parameres (85:1), which are lacking on *Photinus* and *Alychnus* (85:0). *Pseudolychnuris* can be further distinguished from *Alychnus* by the following combination of traits: a proleg bearing one tibial spur (25:1; vs. zero (25:0) in *Alychnus*); and the sternum VIII without a median posterior projection (38:0; present (38:1) in *Alychnus*).

*Pseudolychnuris* is also superficially similar to many species currently listed under *Lucidota*, a “wastebin” genus in need of revision [7]. While it is beyond the scope of this work to elucidate the taxonomy of *Lucidota*, we provide a comparison to the type species, *L. banoni*: the dorsal plate of the phallus in *L. banoni* is entire (i.e., lacks the longitudinal window typical of *Pseudolychnuris*) and lacks the well-developed struts (64:0) seen in *Pseudolychnuris* (64:1); the phallobase is as long as the phallus in *L. banoni* (58:1), but a 1/4 shorter than the phallus in *Pseudolychnuris* (58:0); the subapical spike on the paramere of *Pseudolychnuris* is well-developed and basally elongate, while in *L. banoni* the spike is rudimentary. Together, these traits clearly separate *Pseudolychnuris* and *L. banoni*. 

The spermatophore digesting gland and proctiger could not be determined in the specimen examined, but poor preservation cannot be ruled out. 

**Pseudolychnuris vittata Motschulsky, 1854**

**(** **Figure 3, Figure 4, Figure 5, Figure 6, Figure 7, Figure 8, Figure 9 and Figure 10 and Figure 11E–G)**

*Pseudolychnuris vittata* Motschulsky, 1854: 9 [68]; Olivier, 1911: 70 [63]; Martin et al., 2019: 11 [7].

*Lucidota vittata;* Lacordaire, 1857: 319 [69]; Blackwelder, 1945: 355 [41].

*Alychnus vittipennis* Olivier, 1907: 26 [70].

**Diagnosis:** Color pattern variable (Figure 3 and Figure 10A,B): overall black, except for pronotum, from entirely black to black with expansions pale yellow to orange and elytron, from entirely black to black with an oblique, elongate pale-yellow to orange line. Antennomere III nearly 2× longer than pedicel (Figure 4G,H). Antennal sockets separated by 1/3 labrum greatest width (Figure 4A). Eye small, nearly 1/5 head width (Figure 4A–F).

**Biology:***P. vittata* is a diurnal species commonly seen on flowers of angiosperms native to the Andean paramos, including the “frailejones” (*Espeletia* spp.) (Figure 11E–G). They have been found in copula on these plants (Figure 11E). Interestingly, *P. vittata* has been seen in relatively urban areas of the Meta department, in addition to conservation units on the Paramo. 

**Remarks:***P. vittata* is here regarded as the single species of its genus. *P. vittata* has a remarkably widespread distribution for a species with flightless females. However, other firefly species with similarly flightless females also have widespread distributions, including the type species of the family, *Lampyris noctuluca* L., 1758 (cf. Kazantsev, 2010).

**Material examined:** (♀, UNAB): “COLOMBIA, Cundinamarca, Sopó, 4°44′39″ N 76°56′38″ W, 2580 m, 20 February 1997, al vuelo, M. Becerra cols.//Pseudolychnuris vittate”. (♂, UNAB): “COLOMBIA, Meta, Acacias, Barrio Las Acacias, Carrera 31 Lotes Baldíos, 4°7′6.412′′ N 73°37′51.727′′ W, 26 November 2009, Jama, J. Jiménez cols.//Pseudolychnuris vittate”. (♂, UNAB): “COLOMBIA, Cundinamarca, Anapoima, 4°32′58.26″ N 74°32′7.80″ W, 550 m, 2 September 2011, Captura Manual, En Arbusto, Y. Alonso cols.//Pseudolychnuris vittate”. (♂, UNAB): “COLOMBIA, Valle del Cauca, La Victoria, 4°31′25.751′′ N 76°2′ 9.699′′ W, 12 October 1974, En Maleza, M. Calderón cols.//Pseudolychnuris vittate”. (♂, IAvH-E-219231): “COLOMBIA, Cundinamarca, Guasca, Vereda Rincón del Oso, Finca Suasié, 4°43′36.6″ N 73°51′53″ W, 3400 m, 12 November 2014, Colecta directa en páramo D. Martinéz cols.//Pseudolychnuris vittate”. (2♀, IAvH-E-219232; IAvH-E-219235): “COLOMBIA, Cundinamarca, Guasca, Vereda Rincón del Oso, Finca Suasié, 4°43′36,6″ N 73°51′53″ W, 3400 m, 12 November 2014, Colecta directa en páramo D. Martinéz & K. Pulido cols.//Pseudolychnuris vittate”. (2♀, IAvH): “COLOMBIA, Cundinamarca, Guasca, Vereda Rincón del Oso, Finca Suasié, 4°43′2.1″ N 73°51′58.2″ W, 3090 m, 16 December 2014, Colecta directa en bosque, D. Martinéz cols.//Pseudolychnuris vittate”. (6♀, 7♂, MPUJ), [specimens without data].//“Pseudolychnuris vittate”. (1♀, MPUJ): “COLOMBIA, Cundinamarca, PNN Chingaza, 4300 m//Pseudolychnuris vittate”. (♂, UNAB): “COLOMBIA, Cundinamarca, Fusagasugá, Vereda El Jordán, 4°20′49″ N 74°21′53″ W, 1731 m, 13 September 2001, O. Sequeda & E. Pelayo cols.//Pseudolychnuris vittata”. (♂, ICN-100850): “COLOMBIA, Valle del Cauca, Sevilla, 4°16′1.55″ N 75°55′51.417″ W, 4 February 1974, J. Aguirre cols.//Pseudolychnuris vittate”. (♂, ICN-100862): “COLOMBIA, Cundinamarca, Páramo de Cruz Verde, 4°34′14.987′′ N 74°1′53.03′′W, 17 September 1970, I.S.A cols.//Pseudolychnuris vittate”. (♂, MPUJ): [specimens without data].//“Pseudolychnuris vittate”. (♂, ICN-100863): “COLOMBIA, Caquetá, Florencia, I-69, D. Castro col.//Pseudolychnuris vittate”. (2♀, ICN-100872; ICN-100873): “COLOMBIA, Caquetá, Florencia, I-69, D. Castro col.//Pseudolychnuris vittate”. (♂, IAVH-078002): “COLOMBIA, Valle del Cauca, PNN Farallones de Cali, Anchicaya, 3°26′ N 76°48′ W, 650 m, 28-VIII. 11 September 2001, Malaise, S. Sarria Leg M.2863 col.//Bradley Smith Proj. ‘07 078002//Pseudolychnuris vittate”. (2♂, IAvH-079001, 079002): “COLOMBIA, Cundinamarca, PNN Chingaza, Alto de la Bandera, 4°31′ N 73°45′ W 3660 m, Malaise 8–22 December 2000, E. Niño leg. M1033//Bradley Smith Proj. ‘07 079001//Pseudolychnuris L. SILVEIRA det. 2019. (♂, IAvH-078005): “COLOMBIA, Cundinamarca, PNN Chingaza, Valle del Fraylejon, 4°31′ N 73°45′ W 3170 m, Malaise 31 August–13 September 2000, A. Pérez leg. M732, LAMP & PHENGODIDAE//Bradley Smith Proj. ‘07 078005”. 

***Alychnus* Kirsch, 1865**

**Figure 12:**
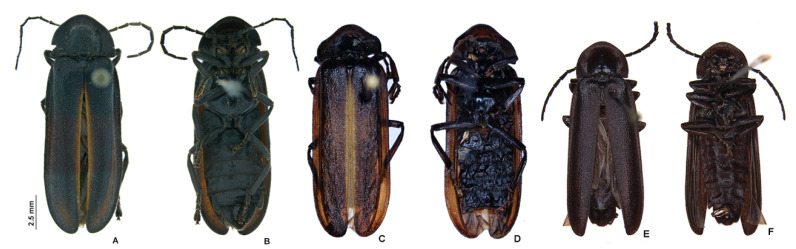
*Alychnus suturalis* (Motschulsky, 1854), males, variation. ♂, IAvH-E-219238, (**A**,**B**): (**A**) dorsal, (**B**) ventral. ♂, MPUJ-0067640, (**C**,**D**): (**C**) dorsal, (**D**) ventral. IAvH-078004 (**E**,**F**): (**E**) dorsal, (**F**) ventral.

**Figure 13:**
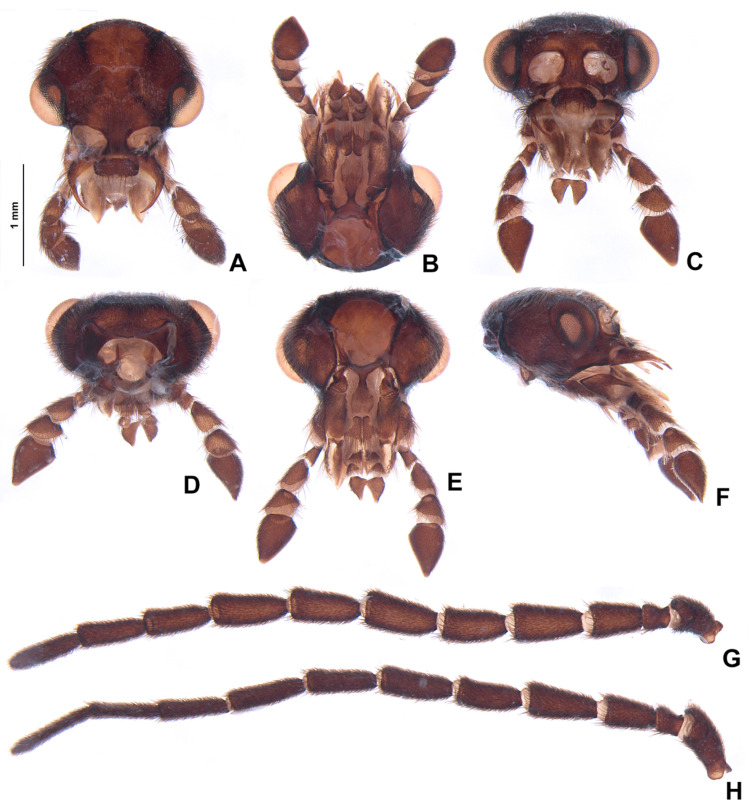
*Alychnus suturalis* (Motschulsky, 1854), male head, IAvH-078004. Head capsule (**A**–**F**): (**A**) dorsal, (**B**) ventral, (**C**) frontal, (**D**) posterior, (**E**) occipital, (**F**) lateral. Antenna (**G**,**H**): (**H**) dorsal, (**G**) lateral.

**Figure 14:**
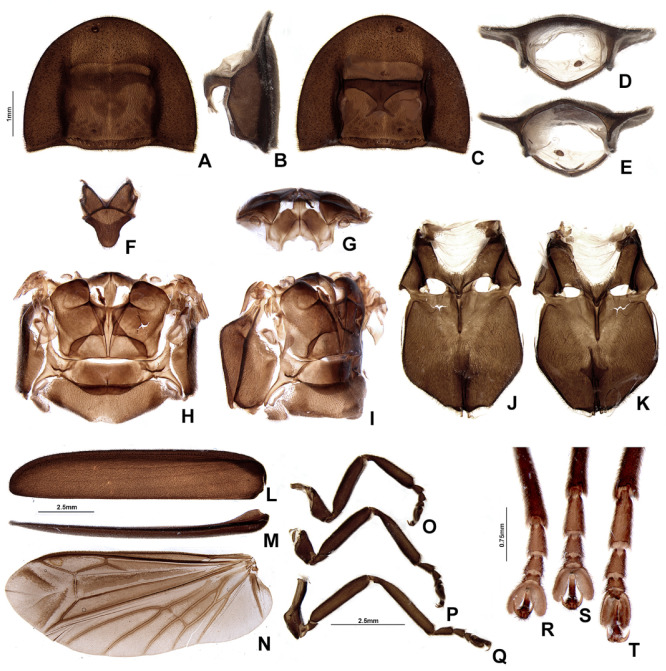
*Alychnus suturalis* (Motschulsky, 1854), male thorax, IAvH-078004. Prothorax (**A**–**E**): (**A**) dorsal, (**B**) lateral, (**C**) ventral, (**D**) anterior, (**E**) posterior. (**F**) Mesoscutellum, dorsal. Metanotum (**G**,**H**): (**G**) anterior, (**H**) dorsal. Pterothorax (**I**–**K**): (**I**) lateral, (**J**) dorsal, (**K**) ventral. Elytra (**L**–**M**): (**L**) ventral, (**M**) lateral. (**N**) Wing, dorsal. Outline of left legs (**O**–**Q**): (**O**) proleg, (**P**) mesoleg, (**Q**) metaleg. Detail of the inwards view of left legs (**R**–**T**), note the tibial spurs: (**R**) proleg, (**S**) mesoleg, (**T**) metaleg.

**Figure 15:**
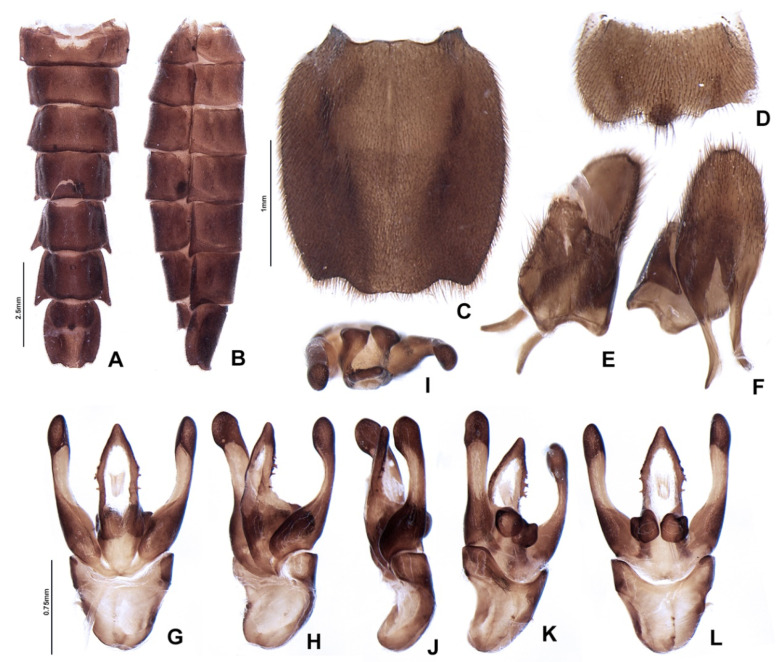
*Alychnus suturalis* (Motschulsky, 1854), male abdomen, IAvH-078004. Whole abdomen (**A**,**B**): (**A**) ventral, (**B**) lateral. (**C**) Pygidium, dorsal. (**D**) Sternum VIII, dorsal. (**E**) Syntergite, ventral. (**F**) Sternum IX, ventral. Aedeagus (**G**–**L**): (**G**) dorsal, (**H**) dorso-lateral, (**I**) anterior, (**J**) lateral, (**K**) ventro-lateral, (**L**) ventral.

**Figure 16:**
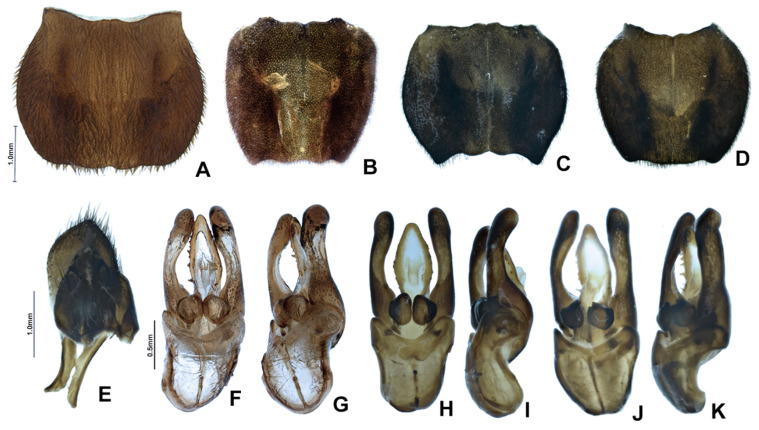
*Alychnus suturalis* (Motschulsky, 1854), male, abdomen variability. Pygidium, dorsal (**A**–**D**): (**A**) ♂, IAvH-E-219238, (**B**) ♂, MPUJ-0067640, (**C**) ♂, MPUJ-0058444, (**D**) ♂, MPUJ-0067638. (**E**) Sternum VIII, ♂, MPUJ-0058444. Aedeagus (**F**–**K**): (**F**,**G**) ♂, IAvH-E-219238, (**H**,**I**) ♂, MPUJ-0058444, (**J**,**K**) ♂, MPUJ-0067638.

**Figure 17:**
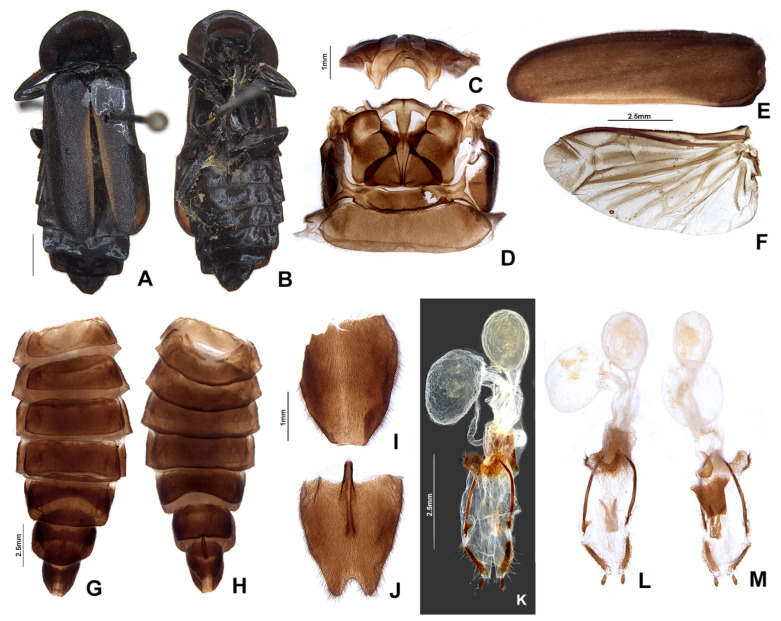
*Alychnus suturalis* (Motschulsky, 1854), ♀ USNM. (**A**) dorsal, (**B**) ventral. Metanotum (**C**,**D**): (**C**) anterior, (**D**) dorsal. (**E**) elytra, ventral, (**F**) wing, dorsal. Whole abdomen (**G**,**H**): (**G**) dorsal, (**H**) ventral. (**I**) Pygidium, dorsal. (**J**) Sternum VIII, (**K**) Internal anatomy of the reproductive tract. Ovipositor (**L**,**M**): (**L**) ventral, (**M**) lateral.

**(Figure 11A–D and Figure 12, Figure 13, Figure 14, Figure 15, Figure 16 and Figure 17)**

*Alychnus* Kirsch, 1865: 71 [71]; Gorham, 1880: 9 [72]; Olivier, 1907: 26 [70].

**Type-species:***Alychnus xanthorraphus* Kirsch, 1865.

**Diagnosis:** Antenna filiform (Figure 13G,H). Labrum with anterior margin straight (Figure 13A), connected to frons by membrane. Pronotum bearing a notch by the posterior angle (Figure 14A). Tibial formula 0-1-1 (Figure 14R–T). Lanterns absent (Figure 15A). Sternum VIII with posterior margin mucronate (Figure 15D). Sternum IX with lateral rods separated (Figure 15F and Figure 16E), basally biconcave. Phallobase mildly emarginate (Figure 15G–L). Dorsal plate hollowed, through which runs the ejaculatory duct, sides bearing spikes (Figure 15G–L). Paramere robust, lacking spikes, inner margin excavate (Figure 15G–L). Female brachypterous (Figure 17A,B), sternum VI with posterior margin emarginate (Figure 17H).

#### 3.2.3. Description

**Male. Head** capsule nearly a 1/3 wider than long (Figure 13A), vertex flat to slightly convex (Figure 13C). Antenna filiform, increasing in length up to antennomere VI, then decreasing toward apex, antennomere III nearly 2× longer than pedicel (Figure 13G,H). Mandibles overlapping (Figure 13C), apex acute. Labrum with anterior margin slightly straight (Figure 13A), connected to frons by membrane throughout. Gula 1/4 as long as submentum, 4× wider than long (Figure 13B). Submentum with anterior margin rounded, sides strongly convergent posteriorly. Occiput ovoid (Figure 13F). Labial palp with sides divergent, apex straight (Figure 13D,F).

**Thorax** with pronotum almost 4× longer than elytron (Figure 12). Pronotum semilunar (Figure 14A), with a notch anterior to the posterior angle; disc convex (Figure 14D,E), lateral expansions almost 1/2 as long as disc and slightly wider than hypomeron depth (Figure 14B,C), anterior expansion 1/2 as long as disc (Figure 5A). Hypomeron nearly 2× longer than deep (Figure 14B). Prosternum with anterior margin almost straight (Figure 14C). Mesoscutellum with posterior margin rounded (Figure 14F). Elytron subparallel-sided, outer margin straight (Figure 14L); marginal costa and epipleuron well developed (Figure 14M). Metanotum with anterior margin bisinuose (Figure 14G), scutum–prescutal ridge reaching posterior margin of alinotum (Figure 14H), metascutellum almost 1/4 as long as alinotum; metapostnotal plate with central 1/3 slightly rounded (Figure 14H). Mesosternum–mesoespisternum suture obliterate (Figure 14J). Hind wing oblong, slightly beyond 2.5× wider than long (Figure 14N), R vein reaching anterior margin, AA3 elongate and at an acute angle with AA4, r3 present, MP_3+4_ split apical to CuA_1_. Pro- and mesoleg without a rounded tooth by the anterior claw. Meso- and metaleg with one tibial spur (Figure 14R–T). Proesdosternum well developed, apically rounded (Figure 14E), metaendosternum diamond-shaped (Figure 14K).

**Abdomen** with sternites II–IX visible (Figure 14A,B). Laterotergite subquadrangular (Figure 14I). Tergites II–VII with posterior angles poorly developed. Lanterns absent (Figure 14B). Pygidium variable (Figure 15C and Figure 16A–D), from slightly wider than long to 2× longer than wide, posterior margin bisinuose, sides rounded to straight, posterior angles rudimentary to well developed and acute. Sternum VIII almost 3× wider than long (Figure 15D), nearly half as long as VII, with posterior margin straight, bearing a median pointed projection, sclerite underneath rudimentary at best. Syntergite longer than wide, and slightly beyond 1/2 sternum IX length, with anterior margin slightly emarginate, posterior corners bearing bristles (Figure 15E). Sternum IX slightly longer than aedeagus (Figure 15E), partially exposed under VIII posterior margin rounded, lateral rods basally divergent, posterior margin rounded to slightly pointed (Figure 16E). **Phallus** with phallobase slightly asymmetric (Figure 8F–H and Figure 15G–L), nearly 1/2 as long as phallus, sides rounded, apical margin slightly emarginate, with an almost complete longitudinal keel. Phallus approximately as long as parameres (Figure 15G–L and Figure 16F–K); dorsal plate lacking basal struts, medially hollowed (interpreted as being clefted, then fused), apex rounded to acute, lateral edged spiked, spikes of variable placement and shape; ventral plate rudimentary, remaining as a sclerite by the opening of the ejaculatory duct; ejaculatory duct running ventrally to dorsal plate (Figure 15G–L); ventrobasal processes present, close-set, globose, sometimes with minute lumps apically (Figure 16J,K). Parameres connected to phallus by membrane (Figure 14G–L and Figure 16F–K), evenly sclerotized, inner margin excavate; co-planar to phallus, rudimentary, acute; subapical spike absent.

**Female.** Elytron brachypterous (Figure 17A,E), nearly 3.5 longer than wide, leaving exposed at least the pygidium, sometimes tergite VI. Hind wing 2× wider than long. metascutellum 1/2 as long as alinotum (Figure 17F). Pygidium 2× longer than wide, sides convergent posteriorly, posterior margin straight (Figure 17I). Sternum VI deeply emarginate (Figure 17H), VII with posterior margin straight, VIII with posterior margin deeply emarginate (Figure 17J), sides convergent posteriorly, spiculum ventrale almost 1/4 as long as sternum. One spermatheca and one accessory gland present, spermatophore digesting gland slightly smaller than spermatheca (Figure 17K). Bursa with a comma-shaped sclerite (Figure 17M). Ovipositor with coxital rods 2× longer than core coxite (Figure 17L,M); proctiger sclerite V-shaped.

**Distribution (based on *A. suturalis*):***Alychnus suturalis* **comb nov.** is endemic to the Colombian Andes, in municipalities in the departments of Boyacá, Cundinamarca, Valle del Cauca, Meta, and Bogota D.C.; being in an altitude range of approximately 1225 to 3773 m.a.s.l. (Figure 10). Therefore, it occurs across the humid and dry high Andean mountains, the sub-humid high Andean mountains, the humid Andean mountains, and the high-Andean plateaus [67]. They occur mostly in protected areas, such as National Natural Parks (PNN) such as PNN Chicaque, SFF Iguaque, and PNN Chingaza. Most specimens studied were collected in September, and no specimen was collected March through July.

**Remarks:***Alychnus* Kirsch, 1865, **stat. rev.** originally included only the type-species: *Alychnus xanthorraphus* Kirsch, 1865, currently a jr. synonym of *Alychnus suturalis* Motschulsky, 1854, **comb. nov.** Here, as per our phylogenetic results, we revalidated *Alychnus* Kirsch, 1865 (see above), to accommodate *A. suturalis* (Motschulsky, 1854). *Alychnuris* is closely related to *Photinus* (Figure 1), but can be distinguished from it by: the tibial formula 0-1-1 (25:0, 27:1, and 28:1) (1-2-2 (25:1, 27:2, and 28:2) in *Photinus*); the globose (66:1) and close-set ventrobasal processes of the dorsal plate (67:1), otherwise transverse (66:0) and divergent (67:0), widely separated in *Photinus*; and the spiked sides of the dorsal plate (78:1), which is smooth in *Photinus* (78:0). A comparison between *Alychnus* and *Pseudolychnuris* is provided at the **Remarks** section of the latter genus.

***Alychnus suturalis* (Motschulsky, 1854) comb. Nov.**

**(** **Figure 11** **A–D and** **Figure 12, Figure 13, Figure 14, Figure 15, Figure 16** **and** **Figure 17** **)**

*Lucidota suturalis* Motschulsky, 1854: 9 [68].

*Alychnus suturalis*; Olivier, 1911: 70 [63].

*Alychnus xanthorraphus* Kirsch, 1865: 72 [71]; Gorham, 1880: 10 [72].

*Lucidota xanthorraphus*; Blackwelder, 1945: 355 [73].

**Diagnosis:** Color pattern overall black, except for: elytron from entirely black to black with sutural line yellow, sometimes expanding outwards (Figure 12 and Figure 17A,B). Antennomere III nearly 2× longer than pedicel (Figure 13G,H). Antennal sockets separated by 1/2 labrum greatest width (Figure 13A). Eye small, nearly 1/5 head width (Figure 13A). 

#### 3.2.4. Description of the Male

**Biology:***A. suturalis* **comb. Nov.** has been found associated with ecosystems with paramo vegetation, being more usual to find them associated with endemic species of this ecosystem such as frailejones (*Espeletia* sp.) (Figure 11A,B). Diurnal females are seen perched on flowers of the *Diplostephium rosmarinifolium* shrub (plant identified by Santiago Guzman, Universidad de Caldas, Colombia) (Figure 11C,D), native to the Andes, that grows in altitude ranges from 2000 to 3900 m.a.s.l. [74], from this behavior it can be assumed that females could feed on the pollen or nectar of these flowers, but more observation of the species in situ is required to understand this behavior. Note that firefly mouthparts, including those of *Alychnus suturalis*, are usually not capable of chewing [75], and are therefore unable to eat solid foods such as pollen. However, a strategy of suspending and sucking pollen should also be considered. Several species of fireflies have been reported to interact with flowers of *Asclepias* in temperate North America [76]. To our knowledge, this is the first report of fireflies interacting with flowers in the tropics.

#### 3.2.5. Remarks

**Material examined:** (♂, IAvH-E-219233): “COLOMBIA, Cundinamarca, Guasca, Vereda Rincón del Oso, Finca Suasié, 4°43′15.2″ N 73°51′50.6″ W, 3310 m, 15 November 2014, Colecta directa en Páramo bajo, D. Martínez col.//*Alychnus suturalis*”. (♂, IAvH-E-219234): “COLOMBIA, Cundinamarca, Guasca, Vereda Rincón del Oso, Finca Suasié, 4°43′36.6″ N 73°51′53″ W, 3400 m, 12 November 2014, Colecta directa en Páramo, D. Martínez & K. Pulido cols.//*Alychnus suturalis*”. (♀, IAvH-E-219236): “COLOMBIA, Cundinamarca, Guasca, Vereda Rincón del Oso, Finca Suasié, 4°43′36.6″ N 73°51′53″ W, 3400 m, 17 December 2014, Colecta directa en Páramo, D. Martínez & K. Pulido cols.//*Alychnus suturalis*”. (2♂, 1♀, IAvH-E-219229; IAvH-E-219237; IAvH-E-219238): “COLOMBIA, Boyacá, SFF Iguaque, Laguna Iguaque, 5°38′ N 73°29′ W, 3340 m, 20 August 1998, Captura manual en Espeletia sp., C. Martínez col.//*Alychnus suturalis*”. (♂, IAvH-E-209188): “COLOMBIA, Boyacá, Arcabuco, Vereda Rupavita, 5°45′14.0″ N 73°22′37.8″ W, 3484 m, 15 October 2018, Captura manual en páramo, A. Lopera & M.I. Castro cols.//*Alychnus suturalis*”. (♀, IAvH-E): “COLOMBIA, Boyacá, Rondón, Vereda Juan Vásquez, 5°24′36.1″ N 73°13′16″ W, 3433 m, 19/23 November 2014, Pitfall, En Páramo, SP. Mondragón, M.I. Castro & J.V Barrera cols.//*Alychnus suturalis*”. (♂, IAvH-E-211950): “COLOMBIA, Boyacá, Tutazá, Vereda Tobal, 6°04′55.7″ N 72°54′59.4″ W, 3773 m, 6 December 2018, Captura manual sobre hojas de frailejón en páramo, M.I. Castro col.//*Alychnus suturalis*”. (♂, IAvH-E-219230): “COLOMBIA, Cundinamarca, La Calera, Vereda Jerusalén, Finca Tierraleja, 4°38′28.68″ N 73°56′1.23″ W, 3500 m, 24 November 2014, Colecta directa en páramo, D. Martínez col.//*Alychnus suturalis*”. (♀, MPUJ): “COLOMBIA, Cundinamarca, PNN Chingaza, 15 May 1989, G. Amat col.//*Alychnus suturalis*”. (♂, MPUJ): “COLOMBIA, Boyacá, Villa de Leyva, SFF Iguaque, Laguna San Rafael, 3600 m, 30 de abril, Colecta durante el día, M. Cuevas & H. Salinas cols.//*Alychnus suturalis*”. (3♀, 1♂, UNAB): “COLOMBIA, Bogotá D.C, 2600 m, 2 December 1973, Colectado en Espeletia sp., M. Proaños col.//*Alychnus suturalis*”. (♂, UNAB): “COLOMBIA, Bogotá D.C, 4°35′56″ N 74°04′51″ W, 2600 m, 4 December 1973, Colectado en Maleza, M. Proaños col.//*Alychnus suturalis*”. (1♂, 1♀ UNAB): “COLOMBIA, Cundinamarca, Tabio, 4°55′1.582′′ N 74°5′48.005′′ W, 12 October 1997, D. Rodríguez col.//*Alychnus suturalis*”. (♂, UNAB): “COLOMBIA, Cundinamarca, Facatativá, Vereda Pueblo viejo, 4°48′59.59″ N 74°20′45.59″ W, 2540 m, 28 August 2011, Captura manual en arbustos cerca de la carretera, Y. Alonso col.//*Alychnus suturalis*”. (♂, UNAB): “COLOMBIA, Valle del cauca, Caicedonia, vía Club de Caza y Pesca, 4°20′0″ N 75°48′0″ W, 1320 m, 22 July 2011, Captura manual, D. Rendón col.//*Alychnus suturalis*”. (♀, UNAB): “COLOMBIA, Cundinamarca, Sasaima, 4°57′59″ N 76°26′15″ W, 1225 m, 26 July 1979, Garavito Ovalle col.//*Alychnus suturalis*”. (10♀, 8♂, CTNI-523; CTNI-3143): “COLOMBIA, Cundinamarca, Chipaque, 4°26′6.573′′ N 74°2′0.739′′ W, 3500 m, 25 September 1941, H. Osorio col.//*Alychnus suturalis*”. (2♀, 1♂, CTNI-523; CTNI-3143): “COLOMBIA, Cundinamarca, Guasca, 4°51′0.563′′ N 73°51′59.961′′ W, 1 November 1939, Colectado en páramo.//*Alychnus suturalis*”. (♂, MPUJ-0067640): “COLOMBIA, Bogotá D.C, 20 June 62.//Alychnus suturalis″. (♂, MPUJ-0058444): “COLOMBIA Meta, Municipio San Juanito, Vereda El Tablón, PNN Chingaza, Sector Páramo de la Silla, 4°27′22′′ N 73°37′34′′ W, 3563 m, 17 March 2018, D. Parrales & G. Fagua cols.//Alychnus suturalis”. (♂, MPUJ-0058390): “COLOMBIA Meta, Municipio San Juanito, Vereda El Tablón, PNN Chingaza, Sector Páramo de la Silla, 4°27′22′′ N 73°37′34′′ W, 3563 m, 17 March 2018, D. Parrales & G. Fagua cols.//Alychnus suturalis”. (♂, MPUJ-0067638): “COLOMBIA, PNN Chingaza.//Alychnus suturalis”. (2♀, USNM): “COLOMBIA, Páramo de Choachi, Cund. 7 August 1965//J. A. Ramos Collector//Alychnus xanthorrapus [sic]”. (♀, USNM): “COLOMBIA, Páramo de Siberia, Cund. 27 February 1965//J. A. Ramos Collector”. (♂, IAvH-078004): “COLOMBIA, Cundinamarca, PNN Chingaza La Siberia, 4°31′ N 73°45′ W 3170 m, Malaise 30 July–10 August 2001, E. Niño leg.//Bradley Smith Proj. ‘07 078004//LAMP-PHENG”. (♂, IAvH-078003): “COLOMBIA, Cundinamarca, PNN Sumapaz Bocatoma, Cerro El Zapato, 3600 m, Malaise 6–20 September 2002, A. Patino leg. M3444//Bradley Smith Proj. ‘07 078003//LAMP-PHENG”.

## 4. Discussion

### 4.1. Convergence on the Páramos 

The fact that *Pseudolychnuris* and *Alychnus* have heretofore been synonyms comes at no surprise. These taxa are indeed strikingly similar in general outline, size, and color patterns, and have largely overlapping distributions. What is more, females of both genera are similarly brachypterous, an observation that has caught the attention of taxonomists from relatively early on [70,71,72]. A rather uncommon phenomenon among diurnal Photinini, female brachyptery was previously reported only for *Pyropyga nigricans* (Say, 1823) [77], *Lucidota luteicollis* (LeConte, 1878) [78], *Phosphaenus* Laporte, 1833, and *Phosphaenopterus* Schaufuss, 1870 [79]. In contrast, brachyptery is somewhat more common in nocturnal *Photinus* species, especially at higher-elevation sites (e.g., *Photinus extensus* Gorham, 1881; [80]), although not unheard of in lower-elevation species (e.g., *P. collustrans* LeConte, 1878 and *Photinus brimleyi* Green, 1956; [81]). In fact, firefly female brachyptery is widespread in the Andean Paramos, including several *Photinus* species, particularly those previously listed under its junior synonym *Macrolampis* Motschulsky, 1853 (e.g., [82]). While the causes for such a trend are yet to be tested, the widespread flightlessness on high-elevation endemics is seen as an adaptation to the strong winds associated with thinner air and lower pressure [83,84]. Therefore, it is likely that *Pseudolychnuris* and *Alychnus* have convergently evolved female brachyptery due to the occurrence in higher elevation sites on the Paramos. However, a sound phylogenetic test for this hypothesis is pending on a better resolved phylogeny, in addition to a broader sampling of female traits across the Photinini. In addition, the similarity in color patterns of these two genera could be the outcome of their participation in similar mimicry rings, although thermal melanism and protection against UV radiation—recurrent themes at higher elevation sites—may also have played a role in shaping these phenotypes. The finding of both species on the same plants (see above) provides a further indication that they participate in the same mimicry ring.

### 4.2. Genitalic Traits and Systematics of the Photinini

The evolution of Photinini seems to be heavily impacted by shifts in signal use [85,86,87,88] . In short, organs involved in emitting (i.e., lanterns) or perceiving (i.e., eyes, antennae) sexual signals have been proposed to be evolutionarily labile, sometimes being significantly diverged among closely related species (e.g., [87]). The present work is congruent with this observation (see Figure 1 and Figure 2). 

Unfortunately, the classification and definition of Photinini genera have been largely built upon differences in these very organs (e.g., [2]), putting in check their definition and calling for a revised classification of this tribe. Meanwhile, other structures involved in reproduction, namely male genitalia, have yielded important characters for discrimination across species and genera [39,60,89,90]. Genitalic traits are of special interest since these are expected to be largely unrelated to changes in the sexual signaling mode, which makes their use especially useful for Photinini taxonomy and the study of their evolution. Female genitalic traits were rarely studied, but also proved successful to discriminate Photinini taxa (e.g., [6,40,79,91]). Nevertheless, the use of genitalic traits to investigate the phylogeny of the Photinini is still in its infancy, although they have yielded important results ([7,13,36]; this work). In fact, nearly half of the characters in this study came from the male genitalia. In contrast, the use of female traits in phylogenetic analyses of Photinini has been hampered by a lack of female data.

A few interesting patterns emerge from the present work, which might impact ongoing and future taxonomic work in Photinini. For example, we found out that both *Alychnus* and *Photinus* share the presence of ventrobasal processes on the dorsal plate of the phallus (char. 65:1; see **Taxonomy** above for a more thorough comparison). The presence of these aedeagal processes has led Zaragoza-Caballero et al. [61] to synonymize *Ellychnia* and *Macrolampis* with *Photinus* in the absence of a phylogenetic treatment, a position kept in the phylogeny of Zaragoza-Caballero et al., [36]. We point out that, despite sharing these ventrobasal processes with *Photinus*, *Alychnus* differs in many other noteworthy traits here, including the shape of these processes themselves, but also: a different configuration of tibial spurs (chars. 25, 27, 28); the unique spiked phallic dorsal plate sides (char. 78); and the phallobase-to-dorsal plate ratio (char. 57), among other traits that have been missing from other phylogenetic treatments. Considering our study, a new look to these particular cases of synonymy is warranted, pending on a more comprehensive dataset—including the type-species of all names involved—analyzed upon a phylogenetic framework. Finally, the function of these ventrobasal processes during copulation is to our knowledge unknown. Observational studies are needed to raise and test hypotheses about the function of these mysterious processes.

## 5. Conclusions

We reviewed and explored the phylogenetic relationships of two Lampyrinae: Photinini genera, *Pseudolychnuris* and *Alychnus* **stat. rev.,** endemic to the Colombian Andes. We found *Alychnus* to be close to *Photinus*, whereas *Pseudolychnuris* affinities remain poorly resolved. Our work provided new characters to diagnose these genera, and also updated distribution maps. 

## Data Availability

The data presented in this study are available in Supplementary Materials.

## Supplementary Materials

The following supporting information can be downloaded at: https://www.mdpi.com/article/10.3390/insects13080697/s1, Supplementary Materials File S1: additional material used in the phylogenetic analyses; Supplementary Materials Table S1: SPR table; Supplementary Materials File S2: Model selection results; Supplementary Materials File S3: character matrix in simplified nexus.
