# Supplementary material for: First Phylogeny of Pseudolychnuris Reveals Its Polyphyly and a Staggering Case of Convergence at the Andean Paramos (Lampyridae: Lampyrini)
Source: Insects. 2022 Aug 3;13(8):697. doi: 10.3390/insects13080697 (PMC9409330; doi:10.3390/insects13080697)
Supplement: Supplementary file 1 [file insects-13-00697-s001.zip › Insects 2022 Supplementary Material I.pdf]

**Supplementary Material I.** Material examined of the outgroup taxa.

**Lampyrinae**

*Costalampys delicata* **Holotype**. **BRAZIL.** *Rio de Janeiro: 1♂, Teresópolis, P.N. Serra dos Órgãos, Malaise trap, PVE9B (22°26’57.8”S, 43°0’13.7”W, 1,236 m), I.2016, L. Silveira col. (DZRJ).*

*Dilychnia guttula*. **BRAZIL**. Acre: Cruzeiro do Sul, Ilha Florianópolis, 1 male, 12–17.V.1981, Norman & Elias (INPA)

*Dadophora hyalina*. **Brazil.** Without other provenance data. 1 Male. (BMNH);

*Dilychnia guttula*. **Brazil.** *Amazonas.* São Paulo Olivença, 10.VIII.1925, 1 female and 1 male, H.L. Boy (MNRJ).

*Ethra marginata*. **Brazil.** *Rio de Janeiro.* Teresópolis, without date, 1 male (DZRJ).

*Haplocauda albertinoi* **BRAZIL**.Acre: Bujari, FES Antimary, 09  20001” S–68  19017” W, 21.x-04. xi, 2016, Malaise grande, E. F. Morato, & J. A. Rafael cols–Rede BIA //HAPLOCAUDA ALBERTINOI HOLOTYPE”.

*Lampyris noctiluca*. **Italy.** Sorrento, 1/VII/1987, J.J.Anderson and Emily Foust col (USNM).

*Lucidota atra*. **United States of America.** NC, Cullowhee, VIII/2020, 1 male (WCCA).

*Lucidota banoni*. **French Guiana.** Without other provenance data, 1 male, 1 female (DZRJ).

*Luciuranus josephi*. **PARATYPES.** BRAZIL. Rio de Janeiro. Teresópolis. PREPVE Pt.5 A 22°27’16.7’’ S 43°01’13.7’’ W 1961m, 11M, 7F, VIII/2014, Silveira & Khattar col. (DZRJ); idem, 1M, 1F, IX/2014, Silveira & Khattar col. (DZRJ); PREPVE Pt.5 B 22°27’18.0’’ S 43°01’12.2’’ W 1949m, 1M, 3F, VIII/2014, Silveira & Khattar col. (DZRJ); PREPVE Pt.6C 22°27’18.0’’ S 43°01’12.2’’ W 2190 m, 2M, 1F, VII/2014, Silveira & Khattar col. (DZRJ); idem, 1F VIII/2014, Silveira & Khattar col. (DZRJ); idem PENSA RIO Pt.5 22°27’18.7’’ S 43°01’32’’ W 1630m, VIII/2013, 17M, 2F R. Monteiro col. (MZSP); idem PENSA RIO Pt.3 22°26’55.6’’ S 43°00’44.3’’ W 1250m, 5M, VIII/2013, R. Monteiro col (MNRJ).

*Luciuranus sinistrus*. **PARATYPES.** BRAZIL**.** Rio de Janeiro. Itatiaia. PENSA RIO Pt.1 22°26’01.4’’, S 44°36’49.3’’ W 1070m, 10M, 15F, X/2014, R. Monteiro col. (DZRJ); PENSA RIO Pt.1 22°26’01.4’’, S 44°36’49.3’’ W 1070 m, Male, X/2014, R.

*Scissicauda disjuncta*. **Brazil.** *Rio de Janeiro.* Teresópolis, Serra dos Órgãos N. P., 1050m, XII/2013, Malaise trap, 1 male and 1 female, R. Monteiro col. (DZRJ)

*Uanauna angaporan*. **PARATYPES. Brazil.** *Rio de Janeiro*, Itaguaí, Parque Estadual Cunhambebe, 22º 50’ 53.4” S 43º 54’ 29.3” W, 150m, IX.2011, 1 male L. Silveira, Clarkson Sampaio & Ferreira-Jr col. (DZRJ); Angra dos Reis, Parque Estadual da Ilha Grande (PEIG), approx. 23º 10’ 30.4’’ S 44º 11’ 11.3’’ W, IX.2008, 2 females, Projeto Coleoptera [J. Mermudes] col. (MNRJ).

*Ybytyramoan praeclarum*. **PARATYPE. Brazil.** *Rio de Janeiro*, Teresópolis, P.N. Serra dos Órgãos, Pedra do Sino, Abrigo 4, 23.IX.2011, 1 male,  N.C., C.L., J.C., Manuella Folly col. (DZRJ).

*Photinus corruscus*. **UNITED STATES OF AMERICA.** North Carolina: Jackson county, Sylva (35.3281198,-83.1756354), VII/2020, 5M, 1F, L. Silveira col. (WCCA)

*Photinus pyralis*. **UNITED STATES OF AMERICA.** North Carolina: Jackson county, Sylva (35.3281198,-83.1756354), VII/2020, 2M, 2F, L. Silveira col.  (WCCA)

*Photinus macdermotti*. **UNITED STATES OF AMERICA.** North Carolina: Jackson county, Sylva (35.3281198,-83.1756354), VI/2022, 2M, L. Silveira col. (WCCA)

*Phosphaenus hemipterus***. PORTUGAL.** Leiria: São de Alge (40°01’22.3’’N, 8°15’47.2’’W), 1M collected by colored pan traps, 23/V/2019; Coimbra: Fajão (40°09’ 54.9’’N, 7°55’22.2’’W), 1M collected by pitfall traps, 19/VI/2019; Seia: Cabeça (Parque Natural da Serra da Estrela), 1M collected by pitfall traps (hosted at Museu de História Natural e da Ciência, University of Porto), 16 to 28-VI-1999; Belgium: Herne, 1M collected by active search, 06/VI/2003, Raphaël De Cock col.

**Incertae sedis**

*Vesta thoracica*. **BRAZIL.** Rio de Janeiro: Guapimirim, Reserva Ecológica de Guapiaçu, XII/2012 1M, L. Silveira col. (DZRJ)
