# Supplementary material for: First Phylogeny of Pseudolychnuris Reveals Its Polyphyly and a Staggering Case of Convergence at the Andean Paramos (Lampyridae: Lampyrini)
Source: Insects. 2022 Aug 3;13(8):697. doi: 10.3390/insects13080697 (PMC9409330; doi:10.3390/insects13080697)
Supplement: Supplementary file 1 [file insects-13-00697-s001.zip › Supplementary Material S2X.pdf]

Table S1. Index values obtained with different K values (according to Mirande, 2009) Trees= number of equally most parsimonious trees obtained; L= number of steps of the most parsimonious tree(s); fit: total fit of the tree; RI= retention index; CI= Consistency index; SPR= average values of SPR comparisons to every other topology.

Interval	F	*K*	Trees	L	fit	RI	CI	SPR
1	50	1.152	2	301	39.309	0,57	0,38	0,92808
2	54	1.353	2	301	37.251	0,57	0,38	0,92808
3	58	1.591	2	301	35.106	0,57	0,38	0,92808
4	62	1.880	2	301	32.843	0,57	0,38	0,92808
5	66	2.237	2	301	30.452	0,57	0,38	0,92808
**6**	**70**	**2.688**	**1**	**301**	**27.912**	**0,57**	**0,38**	**0,92983**
7	74	3.279	1	297	25.162	0,57	0,39	0,88421
8	78	4.085	1	297	22.203	0,57	0,39	0,88421
9	82	5.249	1	297	19.010	0,57	0,39	0,88421
10	86	7.078	1	297	15.534	0,57	0,39	0,88421
11	90	10.370	1	297	11.713	0,58	0,39	0,88421
