# Supplementary figures and images for: First Phylogeny of Pseudolychnuris Reveals Its Polyphyly and a Staggering Case of Convergence at the Andean Paramos (Lampyridae: Lampyrini)
Source: Insects. 2022 Aug 3;13(8):697. doi: 10.3390/insects13080697 (PMC9409330; doi:10.3390/insects13080697)

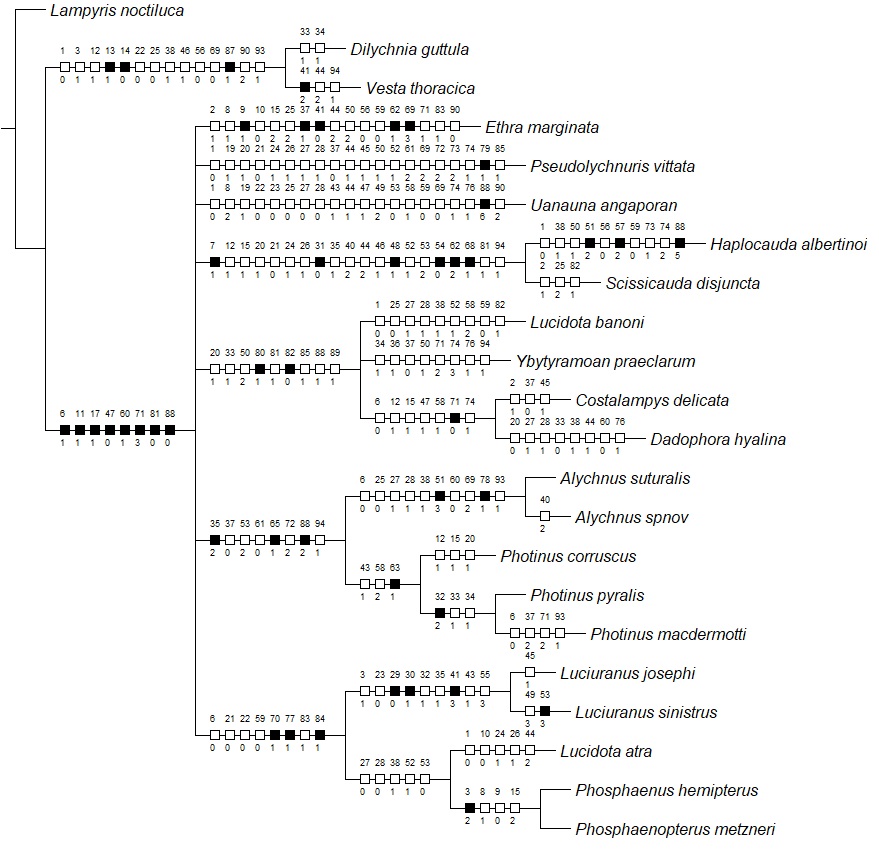

Supplement: Supplementary file 1 [file insects-13-00697-s001.zip › Supp Mat 5 winclada.jpg]
